# S-palmitoylation regulates the function of the mitochondria-associated endoplasmic reticulum membrane to alleviate the senescence of nucleus pulposus cells

**DOI:** 10.1371/journal.pone.0348801

**Published:** 2026-05-22

**Authors:** Qi Liang, Jianfeng Zhang, Jingchuan Yan, Weidong Guo, Jian Zhao, Li Lin, Shuang Li, Daxiong Feng, Bo Liao

**Affiliations:** 1 Advance Spine Surgery Center, Department of Orthopedic Surgery, Tangdu Hospital, The Fourth Military Medical University, Xi’an, Shaanxi, China,‌‌; 2 Shaanxi Institute for Food and Drug Control, Xi’an, Shaanxi, China; 3 Department of Pharmacy, Eighth Hospital of Xi’an City, Xi’an, Shaanxi, China; 4 The Affiliated Hospital of Southwest Medical University, Luzhou City, Sichuan Province, China; The University of British Columbia Life Sciences Institute, CANADA

## Abstract

Intervertebral disc degeneration (IVDD) is the primary cause of spinal degenerative diseases. Nucleus pulposus (NP) cell senescence is a significant pathological manifestation of IVDD. Here, we constructed a hypoxia-induced NP cell model to clarify the mechanisms by which S-palmitoylation is involved in NPC senescence. The IP3R S-palmitoylation of NP cells was significantly reduced under hypoxic conditions, contributing to abnormalities in mitochondria-associated membranes (MAMs). The study found that cellular expression of Bax, Bcl-2, Cleaved-Caspase8, Cleaved-Caspase3, MMP3, and MMP13 was promoted, while COL2 and AGG expression was inhibited. The up-regulated palmitoylation-modifying enzyme DHHC6 can promote IP3R S-palmitoylation modification and regulate GRP75, VDAC1, Drp1, and Mfn2 expression. It can inhibit apoptosis in NP cells, reduce intracellular calcium and ROS levels, elevate mitochondrial membrane potential, and reduce γ-H2AX expression levels. It also inhibited the protein expression levels of hypoxia-induced apoptosis molecules, matrix-degrading enzymes, and up-regulated extracellular matrix protein expression. These results suggested that hypoxia-induced IP3R depalmitoylation might play a role in structural and functional abnormalities in MAMs, which trigger senescence in NP cells.

## 1. Introduction

Lower back pain, a ubiquitous manifestation of degenerative spinal disorders, exerts a profound influence on an individual’s daily life and contributes substantially to the global medical and socioeconomic burden [1]. Intervertebral disc degeneration (IVDD) serves as the pathological cornerstone of spinal degenerative diseases. This pathological alteration frequently coincides with a spectrum of anatomic changes in the peri-intervertebral disc region, encompassing ligamentum flavum hypertrophy, calcification of the posterior longitudinal ligament, and narrowing of the neurapiphyseal canals [2]. Furthermore, it may coexist with posterior articular degeneration. It is imperative to emphasize that these assessments are objective in nature, devoid of subjective biases. The degenerative spinal conditions stemming from IVDD have emerged as a significant global medical and socioeconomic challenge [3,4]. The intervertebral disc comprises the central nucleus pulposus (NP), the annulus fibrosus (AF), and the superior and inferior cartilaginous endplates. Degenerative alterations typically initiate within the NP, leading to decreased water content, cellular and metabolic dysregulation, and precipitating a cascade of events that escalate lesions and ultimately culminate in a range of clinical manifestations [5]. Currently, clinical interventions for IVDD primarily focus on mitigating symptoms rather than halting, slowing, or reversing the pathological progression of the disease. Moreover, there is a notable absence of effective preventive or curative strategies. Consequently, it is paramount to vigorously pursue more efficacious treatments and elucidate their underlying therapeutic mechanisms. Additionally, the identification of sensitive and specific biomarkers at the molecular level holds promise for advancing early diagnosis, treatment, and prognosis of IVDD.

Although the precise pathogenesis of IVDD remains elusive, it is widely regarded as a natural consequence of disc aging [6]. Cellular senescence within the NP is a pivotal pathological feature of IVDD. Hypoxic stress in the intervertebral disc tissues induces an elevation in reactive oxygen species (ROS) levels, mitochondrial dysfunction, and a decline in cellular metabolism, which collectively contribute to cellular senescence and apoptosis. Furthermore, the impaired functionality of senescent cells further intensifies the hypoxic conditions within the disc tissue [7]. DHHC6 is a palmitoyltransferase that undergoes S-palmitoylation via three cysteine residues (Cys-328, Cys-329, and Cys-343) located on its SH3_2 domain [8]. Palmitoylation represents a reversible post-translational modification that profoundly influences protein localization, diffusion, and stability [9]. Our study uncovered that the palmitoylation of DHHC6 is regulated by the upstream palmitoyltransferase ZDHHC16, thereby elucidating the existence of a novel palmitoylation cascade reaction. Additionally, the depalmitoylation process of DHHC6 is facilitated by acyl protein thioesterase Lysophospholipase A2 (APT2). Utilizing specific site mutations, experimental kinetic parameter measurements, and data-driven mathematical modeling, researchers gained a comprehensive understanding of the eight distinct palmitoylated species of DHHC6. These species undergo rapid transitions facilitated by ZDHHC16 and APT2, with each species exhibiting unique conversion rates and activities. This finding allows cells to dynamically regulate their DHHC6 activity, highlighting the complexity and flexibility of palmitoylation-mediated regulatory mechanisms in IVDD.

The disruption of the structural integrity of mitochondria-associated membranes (MAMs) is implicated in cellular senescence. However, it is unclear whether it mediates senescence in IVDD NP cells. S-palmitoylation is a post-translational lipid modification of proteins. It has been found that inositol 1,4,5-trisphosphate receptor (IP3R) S-palmitoylation is involved in the regulation of calcium fluxes and immune activation [10]. While IP3R is responsible for the structural composition of MAMs and maintaining their functional homeostasis, the calcium pathway, which includes IP3R-Grp75-VDAC, plays a crucial role in maintaining mitochondrial homeostasis [11]. This project aims to investigate the regulation of IP3R S-palmitoylation modification in the hypoxic microenvironment, its effects on the structure and calcium ion transfer function of MAMs, and whether it is a key factor in the disruption of mitochondrial homeostasis that triggers NP cell senescence.

## 2. Materials and methods

### 2.1. Cell culture

The human intervertebral disc NP cells were purchased from Wuhan Ponosi Life Technology Co., Ltd. The NP cells were cultured in a 37°C, 5% CO_2_ cell culture incubator using human intervertebral disc NP cell complete medium culture (CM-H097). The medium was changed every two days. The experimental NP cells were between the 3rd and 6th generations.

The Control group was cultured under normal conditions, while the HI group was created by incubating cells in 1% O_2_, 94% N_2_, and 5% CO_2_ for 6 hours under hypoxic conditions. The OE-NC+HI group was induced by hypoxia and transfected with the OE-NC plasmid, while the OE-DHHC6+HI group was induced by hypoxia and transfected with the OE-DHHC6 plasmid. The OE-NC+HI group and OE-DHHC6+HI group were constructed by constructing the OE-DHHC6 lentivirus overexpression vector (synthesized by Biotech (Shanghai) Co., Ltd.) and selecting the pLV Puro vector. Obtain the DHHC6 sequence from NCBI, insert DHHC6 into the pLV Puro vector, and construct the pLV Puro-OE-DHHC6 plasmid. Packaging it into a lentivirus using a lentivirus packaging system, provided by Sangon Biotech (Shanghai) Co., Ltd. After obtaining the constructed lentiviral expression vector, it was transfected into human intervertebral disc NP cells.

### 2.2. Detection of apoptosis by flow cytometry

Detection of apoptosis in nucleus pulposus (NP) cells was performed using flow cytometry. Cells were seeded at a density of 1 × 10⁶ cells/well in 6-well plates and cultured to form a confluent monolayer, which was then trypsinized to create a single-cell suspension. Subsequently, 5 μL Annexin V-FITC and 5 μL PI solution were added, followed by 15 minutes of incubation in the dark. Finally, 400 μL of 1 × Annexin V Binding Solution was introduced, and the sample was analyzed within 1 hour using flow cytometry.

### 2.3. Fluo-4 AM assay for cellular calcium ion concentration

Cells were seeded at a density of 1 × 10⁷ cells per well in a 6-well plate, with 1 mL of complete culture medium added to each well. After 24 hours of incubation, the medium was carefully aspirated, and 0.5 μM Fluo-4 AM calcium ion indicator dissolved in serum-free medium was added. The cells were then incubated for 20−30 minutes at room temperature in the dark, followed by three washes with Dulbecco’s Phosphate Buffered Saline (DPBS) (without Mg^2+^ and Ca^2+^). Fluorescence was immediately visualized using a fluorescence microscope, and images were rapidly captured for subsequent analysis with ImageJ software. The calcium response of each group of images is quantified as ΔF/F_0_, where F_0_ is the average baseline fluorescence intensity of the Control group, F_P_ is the real-time fluorescence intensity, and ΔF is F_P_-F_0_. For the detection of baseline ER Ca^2+^ concentration, the Fluo-4 AM fluorescence intensity of cells was quantified immediately after three washes with DPBS (before thapsigargin addition) as the baseline value; then thapsigargin (1 μM) was added to the cells, and the fluorescence intensity was detected again after 30 min to analyze the ER Ca^2+^ content following SERCA inhibition.

### 2.4. Intracellular ROS assay

Experiments were conducted utilizing the ROS detection kit. NP cells were seeded in culture dishes to achieve a confluency of 50–70%. DCFH-DA was diluted in serum-free culture medium at a 1:1000 ratio to reach a final concentration of 10 μM. The diluted DCFH-DA was added to cover the cells, and fluorescence was promptly detected using a fluorescence microscope.

### 2.5. Detection of mitochondrial membrane potential by JC-1 staining

NP cells were carefully seeded into 6-well plates at a density of 1 × 10⁴ cells per well. After ensuring even distribution, 1 mL of JC-1 staining solution was gently added to each well. The plates were then incubated at 37 °C for 20 minutes to allow dye uptake. Subsequently, 2 mL of serum- and phenol red-containing cell culture medium was added, and the cells were observed under a fluorescence microscope.

### 2.6. Percoll centrifugation for separation of MAMs

After different culture treatments, approximately 2 × 10⁹ nucleus pulposus (NP) cells were harvested, resuspended in pre-chilled buffer, and manually homogenized on ice at 4°C, with all operations performed in an ice bath throughout to prevent protein degradation. The homogenate was centrifuged at 740 × g for 5 min at 4°C, repeated twice to remove nuclei and tissue debris, and the supernatant was collected. The supernatant was then centrifuged at 9000 × g for 30 min at 4°C, and separated into supernatant fraction and crude mitochondria pellet for respective purification. For the supernatant fraction, it was centrifuged at 20000 × g for 60 min at 4°C to discard the pellet, and the resulting supernatant was ultracentrifuged at 100000 × g for 60 min at 4°C to obtain purified cytosol (pCyto) and endoplasmic reticulum (ER) fractions. For the crude mitochondria pellet, it was resuspended and layered on 30% Percoll medium, followed by ultracentrifugation at 95000 × g for 30 min at 4°C to isolate crude MAM and pure mitochondrial fractions. Both fractions were further purified by centrifugation at 9000 × g for 30 min at 4°C: the pellet of pure mitochondrial fraction was collected as purified mitochondria (pMito), while the supernatant of crude MAM fraction was ultracentrifuged at 100000 × g for 60 min at 4°C, with the resulting pellet as purified MAM fraction [12]. All fractions were snap-frozen in liquid nitrogen and stored at −80°C. The separation purity was verified by Western blot of specific marker proteins, which showed that MAM fraction was enriched with both Calnexin and COX IV, while pMito was only specifically enriched with COX IV without detectable Calnexin residue, confirming satisfactory separation efficiency.

### 2.7. Western blot detection of protein expression

Total protein was extracted with RIPA lysate, and protein quantification was performed by the BCA method. Proteins were separated by SDS-PAGE gel electrophoresis and transferred to a PVDF membrane. The membrane was then immersed in 5% skimmed milk for 2h at room temperature, incubated overnight at 4°C with appropriate primary antibodies (Table 1), and then incubated with secondary antibodies. Membranes were developed using a chemiluminescence imaging system and analyzed in grey scale using ImageJ software.

**Table 1:** Antibodies were diluted as follows.

Antibodies	Dilution (application)	
IP3R	1:1000 (WB)	
Bax	1:1000 (WB)	
Bcl-2	1:1000 (WB)	
Cleaved-Caspase8	1:1000 (WB)	
Cleaved-Caspase3	1:1000 (WB)	
COL2	1:1000 (WB)	
AGG	1:1000 (WB)	
MMP3	1:2000 (WB)	
MMP13	1:3000 (WB)	
GRP75	1:1000 (WB)	
VDAC1	1:1000 (WB)	
Drp1	1:1000 (WB)	
Mfn2	1:5000 (WB)	
GAPDH	1:50000 (WB)	
COX IV	1:2000 (WB)	
Goat Anti-Mouse IgG H&L(HRP)	1:20000 (WB)	
Goat Anti-Mouse IgG H&L(HRP)	1:20000 (WB)	
GRP78	1:1000 (WB)	
CHOP	1:1000 (WB)	
XBP1s	1:1000 (WB)	

### 2.8. Transmission electron microscopy experiments

NP cells were fixed by first incubating with 1 mL of 2.5% glutaraldehyde for 1 hour, followed by a secondary fixation with 0.5 mL of 1% osmium tetroxide (referred to here as “starvation acid” corrected for clarity) for 1 hour. After rinsing with PBS, samples were dehydrated, embedded, and sectioned to 70 nm thickness. Sections were stained with lead citrate for 10 minutes and uranyl acetate for 30 minutes before imaging via transmission electron microscopy at 40,000–80,000 × resolution to analyze MAM counts and mitochondrial/ER morphology. At the same time, the distance between the ER and mitochondria within each cell was quantitatively analyzed using the software ImageJ. Six replicates were measured for each group for statistical analysis.

### 2.9. Acyl-biotinyl exchange

NP cells were harvested and lysed using a specialized lysis buffer to extract total proteins. Protein concentration was determined using the BCA protein assay kit. N-Ethylmaleimide (NEM) was added to block free thiols, and the solution was concentrated by centrifugation. Hydroxylamine (HAM) was used to cleave thioester bonds, followed by another concentration step. HPDP-Biotin was then added for biotinylation of free thiols, and the mixture was incubated with streptavidin-coated magnetic beads for enrichment. Finally, Western Blot analysis was performed to detect and label palmitoylated proteins.

### 2.10. β-galactosidase staining

Inoculate 1 × 10^6^ cells per well of logarithmic phase growth cells into a 6-well plate, divide the cells into groups, and culture them in a 37 °C, 5% CO2 cell culture incubator. Remove the supernatant from the 6-well plate, wash it once with PBS, add 1 mL of β-galactosidase staining fixative, and fix at room temperature for 15 minutes. Remove the cell fixative and wash these cells three times with PBS for 3 minutes each time. Remove PBS and add 1 milliliter of staining solution to each well. Incubate overnight at 37 °C and seal the 6-well plate with cling film to prevent evaporation. Observe under a regular optical microscope.

### 2.11. Ki-67 staining

Inoculate cells in the logarithmic growth phase into a 6-well plate with 1 × 10^6^ cells per well, and treat them according to grouping. Remove the discarded supernatant from the 6-well plate, gently wash with PBS three times, add 1 ml of 4% paraformaldehyde, and fix the cells on a shaker (50 rpm) for 20 minutes. Discard the supernatant, gently wash with PBS three times, add 1 ml of 0.25% Triton-X100, and let it permeate on a shaker (50 rpm) for 20 minutes. Gently rinse with PBS three times, add 1 ml goat serum, and block for 30 minutes. Remove the blocking solution, add 1 ml of primary antibody (1:200), and incubate overnight at 4 °C. Retrieve the primary antibody and wash 3 times with TBST for 5 minutes each time. Add 1 ml of secondary antibody (1:500) and incubate on a shaker (50 rpm) at room temperature in the dark for 1 hour. Discard the secondary antibody and wash with TBST three times, each time for 5 minutes. After staining with DAPI for 5 minutes, remove DAPI and wash 3 times with TBST for 5 minutes each time. Fluoroshield containing 4,’6-diamino-2-phenylindole (DAPI) ^®®TM^ Fix the glass slide and take a photo under a fluorescence microscope. Use ImageJ (National Institutes of Health, NIH) to analyze fluorescence intensity.

### 2.12. Data analysis

The data were analyzed and graphed using GraphPad Prism 9 (Version 9.4.0). All data were expressed as means ± SD. Statistical differences between groups were tested using the T-test or One-way test, with P-values less than 0.05 considered significant.

## 3. Results

### 3.1. Effect of overexpression of DHHC6 on NP cells

**Fig 1:**
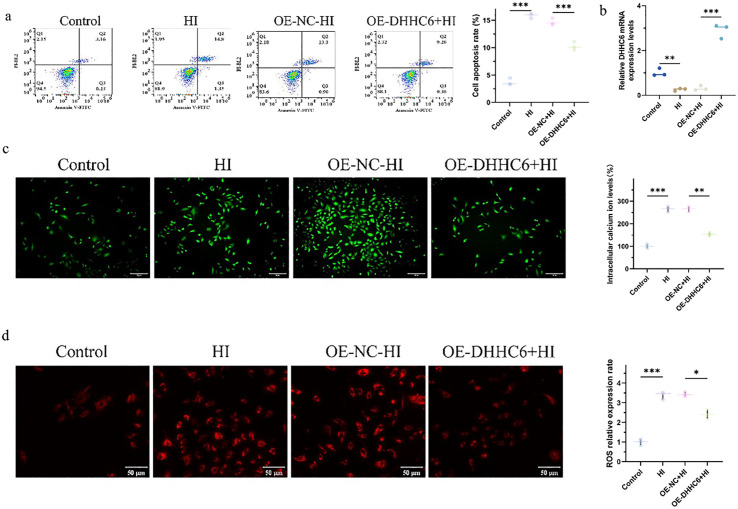
Detection of apoptosis, calcium, and ROS levels. (a) Flow detection of apoptosis levels in each group. (b) The mRNA expression of DHHC6 was detected by RT-qPCR. (c) Fluo-4 AM fluorescent probe for calcium levels in each group. (d) ROS fluorescent probe to detect ROS level. ^*^p < 0.05，^**^p < 0.01，^***^p < 0.001.

**Fig 2:**
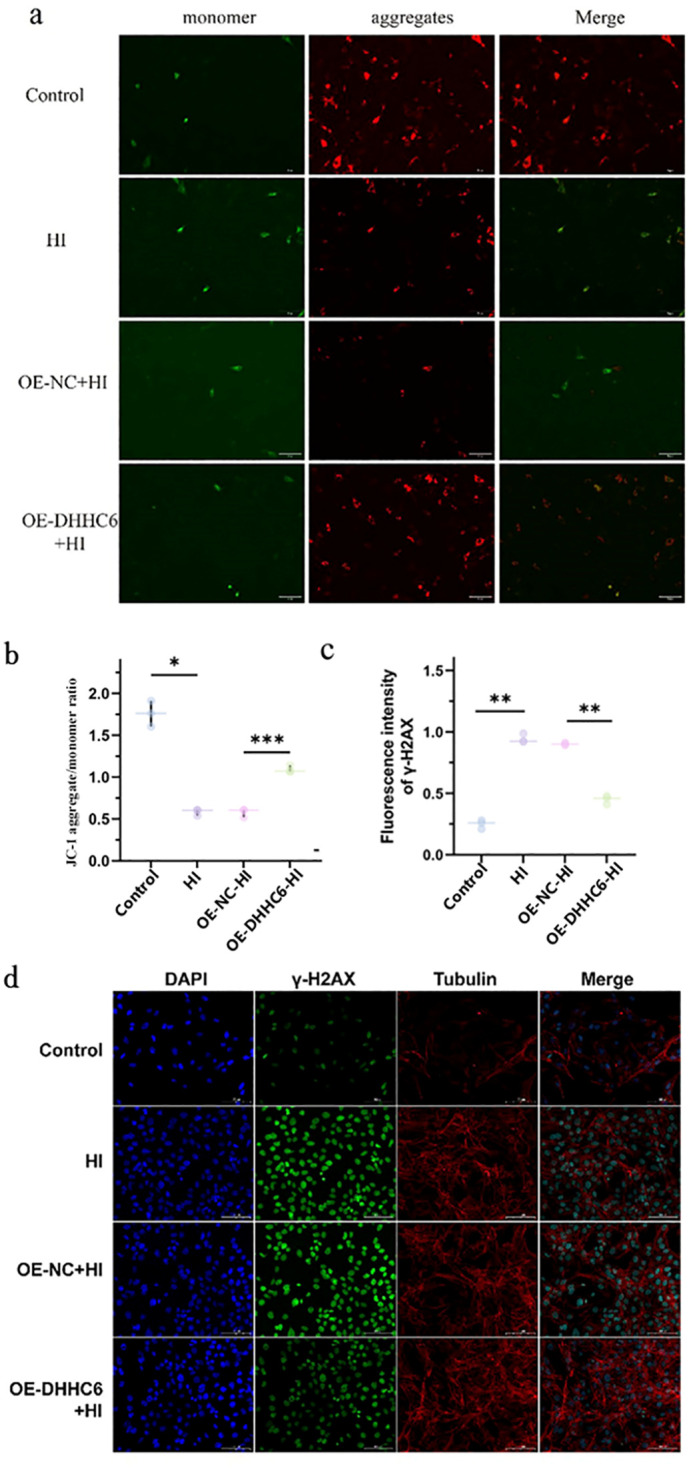
Detection of cellular mitochondrial membrane potential and γ-H2AX expression levels. (a) Mitochondrial membrane potential of each group of cells was detected by the JC-1 kit. (b) Statistical analysis of mitochondrial membrane epotential of each group of cells detected by the JC-1 kit. (c) Statistical analysis of immunofluorescence staining for detecting the expression level of γ-H2AX. (d) Immunofluorescence staining to detect the expression level of γ-H2AX. ^*^p < 0.05，^**^p < 0.01，^***^p < 0.001.

As illustrated in Fig 1, hypoxia triggered apoptosis in NP cells (Fig 1a), accompanied by elevated intracellular calcium ion levels (Fig 1c) and increased ROS production (Fig 1d). RT-qPCR analysis demonstrated a significant reduction in DHHC6 mRNA expression in the hypoxia-induced (HI) group compared to the Control group. Conversely, overexpression of DHHC6 (OE-DHHC6 + HI group) led to a marked increase in DHHC6 mRNA levels relative to the OE-NC + HI group (Fig 1b). Furthermore, hypoxia induced a decrease in mitochondrial membrane potential (Figs 2a and 2b) and upregulated the expression of the aging phenotype marker γ-H2AX (Figs 2c and 2d). Transfection with the DHHC6-NC plasmid did not alter the phenotype or protein molecular profile of NP cells. Taken together, our results suggested that overexpression of DHHC6 effectively decreased NP cell apoptosis, reduced intracellular calcium ion and ROS levels, restored mitochondrial membrane potential, and diminished γ-H2AX expression.

### 3.2. The effect of DHHC6 on cellular aging

**Fig 3:**
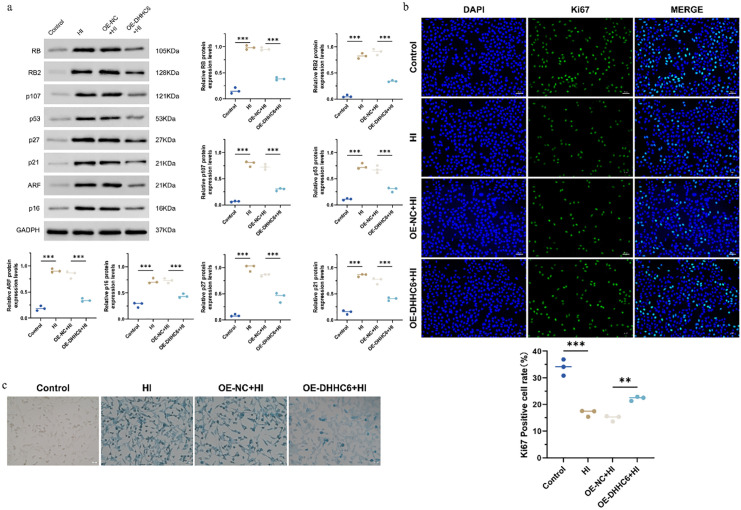
Detection of cellular aging indicators. (a) Western Blot was used to detect RB, RB2, p107, p53, p27, p21, ARF, and p16 to evaluate the details of each group. Changes in cellular aging. (b) Observation of cellular aging through Ki-67 staining. (c) Observation of cellular aging by β-galactosidase staining. ^*^p < 0.05，^**^p < 0.01，^***^p < 0.001.

To assess the impact of DHHC6 on cellular aging, we performed Western Blot analysis, which revealed a notable upregulation of aging-related protein expression in the HI group compared to the Control group. Conversely, the OE-DHHC6 + HI group resulted in a significant reduction of these aging-related protein levels relative to the OE-NC + HI group (Fig 3a). This reduction suggested that the S-palmitoylation catalyzed by DHHC6 may be a factor in alleviating cellular aging. Additionally, Ki-67 immunostaining demonstrated a marked decrease in the Ki-67 positivity rate in the HI group compared to the Control group, while the OE-DHHC6 + HI group exhibited a significant increase in Ki-67 positivity compared to the OE-NC + HI group (Fig 3b). This difference in Ki-67 indicated the potential role of DHHC6 in maintaining NP cell proliferation activity. Furthermore, β-galactosidase staining indicated intensified β-gal staining in the HI group compared to the Control group, whereas the OE-DHHC6 + HI group displayed notably lighter β-gal staining compared to the OE-NC + HI group (Fig 3c). The results of β-gal staining also indicated the promising role of DHHC6 in NP cell aging.

### 3.3. Verification of the purity of subcellular component separation

All target marker proteins were detected in the whole cell lysate, confirming the favorable integrity of protein samples and the reliability of the antibody detection system. Western blot was performed to verify the purity of the isolated mitochondria-associated membrane (MAM) fraction via detecting subcellular-specific marker proteins in whole cell lysate, purified cytosol (pCyto), pure mitochondria (pMito), MAM and purified endoplasmic reticulum (ER) fractions. The results showed that all target proteins were detected in whole cell lysate, confirming the validity of the detection system. pCyto, pMito and ER fractions were specifically enriched with their corresponding markers (Tubulin for pCyto; COX IV and GRP75 for pMito; Calnexin, GRP78 and IP3R for ER) without cross-contamination, verifying the reliability of our isolation protocol (S1 Fig). The MAM fraction was simultaneously enriched with both ER markers (Calnexin, GRP78) and mitochondrial markers (COX IV, GRP75), consistent with the structural characteristics of MAM as the mitochondria-ER contact site (S2 Fig). No cross-contamination from cytosol or free organelles was detected in the MAM fraction, collectively confirming that high-purity MAM was successfully isolated in this study.

### 3.4. Effect of overexpression of DHHC6 on MAMs

**Fig 4:**
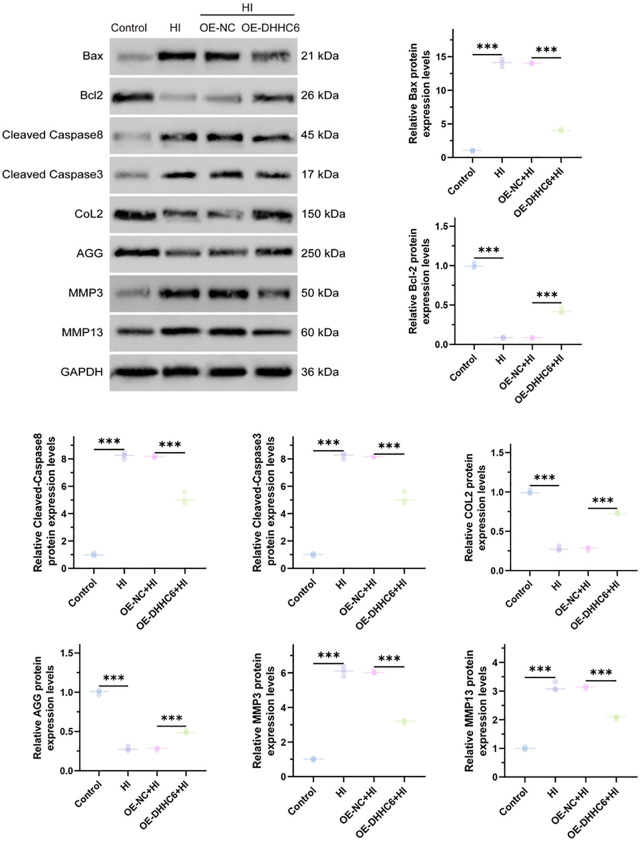
Western Blot detection of Bax, Bcl-2, cleaved Caspase8, cleaved Caspase3, Col2, AGG, MMP3, MMP13 protein expression levels. ^***^p < 0.001.

**Fig 5:**
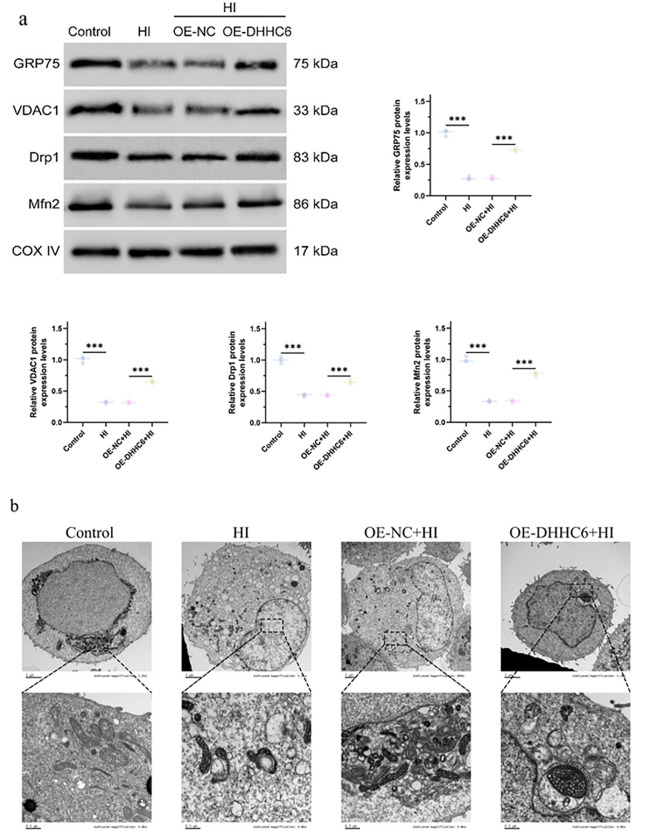
Detection of protein expression levels and observation of cellular microstructure. (a) Percoll centrifugation to isolate subcellular structural MAMs, and Western Blot to detect GRP75, VDAC1, Drp1, and Mfn2 protein expression levels. ^***^p < 0.001. (b) TEM observation of cell microscopic morphology.

As depicted in Fig 4, Western Blot analysis was employed to detect the expression levels of Bax, Bcl, Cleaved-Caspase8, Cleaved-Caspase3, COL2, AGG, MMP3, and MMP13 in NP cells of intervertebral discs across various groups. The results demonstrated that hypoxia induced an upregulation of apoptosis-related molecules and matrix-degrading enzyme proteins, while concurrently suppressing the expression of extracellular matrix proteins. Overexpression of DHHC6 effectively reversed the protein expression patterns of hypoxia-induced apoptosis-related molecules, extracellular matrix components, and matrix-degrading enzymes. Subsequently, functional proteins within the mitochondrial-associated membranes (MAMs), namely GRP75, VDAC1, Drp1, and Mfn2, were assessed by Western Blot following isolation of subcellular MAMs via Percoll centrifugation (Fig 5a). We evaluated the Calnexin and PDIA3 protein expression levels using WB analysis.

### 3.5. Analysis by electron microscopy

Transmission electron microscopy (TEM) was performed to examine the ultrastructural changes of cells in each group (Fig 5b). Cells in the normal control group showed intact structure with regular nuclei, continuous and clear nuclear membrane, evenly distributed chromatin, structurally intact mitochondria with well-arranged inner cristae, and physiological flattened cystic endoplasmic reticulum (ER) with clear membrane boundary, without ischemic-hypoxic organelle damage. Cells in the hypoxia-ischemia (HI) model group exhibited typical ischemic-hypoxic injury, including extensive cytoplasmic vacuolization, pyknotic irregular nuclei with impaired nuclear membrane integrity, markedly swollen mitochondria with disorganized or absent inner cristae, dilated ER with blurred boundary, and significantly increased abnormal contact sites of mitochondria-associated ER membranes (MAMs), indicating severe disruption of organelle structural and functional homeostasis. No significant difference in ultrastructural damage severity was observed between the overexpression negative control + HI group (OE-NC+HI) and the HI model group, confirming that negative control transfection had no specific intervention effect on HI-induced cellular ultrastructural damage. In contrast, the DHHC6 overexpression + HI group (OE-DHHC6+HI) showed markedly alleviated ultrastructural damage compared with the OE-NC+HI group, with intact cell contour, fully restored normal morphology of nuclei, mitochondria and ER, and significantly reduced abnormal MAM contact sites. Collectively, these results demonstrate that DHHC6 overexpression effectively maintains normal ER morphological structure under hypoxia-ischemia and mitigates HI-induced organelle ultrastructural damage.

To investigate the regulatory effect of DHHC6 overexpression on the spatial crosstalk between the endoplasmic reticulum (ER) and mitochondria under hypoxia-ischemia (HI) injury, we quantitatively measured the inter-organelle distance between ER and mitochondria in cells subjected to different treatments, with the results presented in the box plot. Compared with the normal control group, HI treatment triggered an extremely significant increase in ER-mitochondria distance (P < 0.001), with the median distance rising from approximately 18 nm in the control group to around 52 nm in the HI group, indicating that HI exposure severely disrupted the physiological tight juxtaposition between ER and mitochondria. Meanwhile, transfection with the overexpression negative control (OE-NC) failed to reverse the abnormal enlargement of ER-mitochondria distance induced by HI. No significant difference was observed in ER-mitochondria distance between the OE-NC+HI group and the HI group, and both were extremely significantly higher than that of the normal control group (P < 0.001, 3S Fig). In contrast, DHHC6 overexpression markedly reversed the abnormal elevation of inter-organelle distance caused by HI injury. The median ER-mitochondria distance in the OE-DHHC6+HI group was reduced to approximately 28 nm, which was extremely significantly lower than that in the OE-NC+HI group (P < 0.001, 3S Fig), and was largely restored to a level close to the physiological baseline. Collectively, these results demonstrate that HI injury significantly impairs the spatial juxtaposition between ER and mitochondria, while DHHC6 overexpression effectively antagonizes HI-induced abnormal spatial structure of organelles and preserves the physiological interaction space between ER and mitochondria, providing direct organelle-level experimental evidence for the protective role of DHHC6 against HI injury.

### 3.6. Effect of overexpression of DHHC6 on IP3R S-palmitoylation modifications

**Fig 6:**
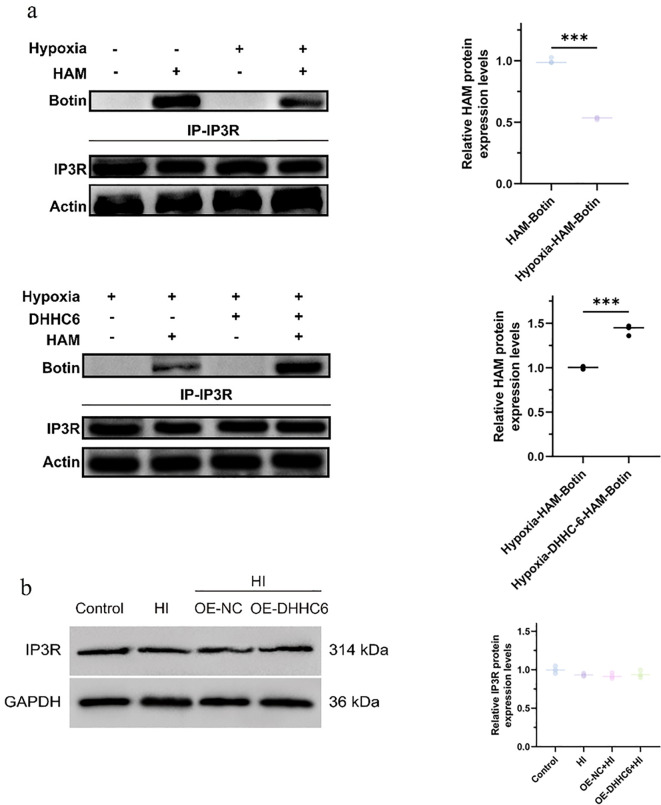
Detection of IP3R S-palmitoylation modification and protein expression levels. **(a)** ABE and Co-IP detect IP3R S-palmitoylation modification levels. **(b)** Western Blot detection of IP3R protein expression level. ^***^p < 0.001.

As shown in Fig 6a, the results of the ABE method and Co-IP experiments indicate that hypoxia reduces the level of IP3R S-palmitoylation modification. Overexpression of DHHC6 promotes IP3R S-palmitoylation. Subsequently, we detected the IP3R expression level by Western blot (Fig 6b). Neither hypoxia-induced nor overexpression of DHHC6 treatment of intervertebral disc NP cells did not affected their IP3R protein expression level.

### 3.7. Detection of Ca^2+^ levels in ER

To elucidate the regulatory effect of DHHC6 on ER calcium homeostasis under HI injury, we further detected ER Ca^2+^ levels in cells subjected to different treatments. As shown in panel A, there was no statistically significant difference in baseline ER Ca^2+^ fluorescence intensity among the Control, HI, OE-NC+HI and OE-DHHC6+HI groups before thapsigargin treatment (P > 0.05, 4S Fig), indicating that HI exposure, negative control transfection and DHHC6 overexpression did not exert a marked effect on resting basal ER Ca^2+^ levels in cells. After thapsigargin administration to inhibit SERCA activity and deplete ER Ca^2+^ stores, we found that ER Ca^2+^ content in the HI group was extremely significantly reduced compared with the Control group (P < 0.001, 4S Fig), suggesting that HI injury severely impaired ER calcium storage capacity and induced ER calcium homeostasis imbalance in cells. The ER Ca^2+^ content in the OE-NC+HI group showed no obvious difference from that in the HI group, which remained at an extremely low level and was significantly lower than that in the Control group (P < 0.001, 4S Fig). In contrast, DHHC6 overexpression markedly reversed HI-induced depletion of ER Ca^2+^ stores, and ER Ca^2+^ content in the OE-DHHC6+HI group was extremely significantly upregulated compared with the OE-NC+HI group (P < 0.001, 4S Fig), effectively restoring the ER calcium storage capacity of cells under HI insult. Collectively, these results confirm that DHHC6 overexpression effectively preserves ER calcium homeostasis under HI injury, and its protective effect is not mediated by altering basal ER Ca^2+^ levels, but by antagonizing HI-induced impairment of ER calcium storage capacity.

## 4. Discussion

The cause of IVDD is an abnormal process of cellular degeneration that is related to age and genetics. This process can be accelerated by toxic factors, nutrient availability, and mechanical stress [11]. The intervertebral disc is in a microenvironment of hypoxia, avascularity, and lactic acid accumulation. The poor self-renewal of damaged tissue accelerates the progression of disc degeneration [12]. The NP comprises NP cells and extracellular matrix components. The normal function and number of NP cells are essential for maintaining metabolic homeostasis of the extracellular matrix, stability of the intervertebral disc environment, and mechanical function [13]. Senescence and apoptosis of NP cells decrease the extracellular matrix synthesis and change its composition, leading to an increase in pressure on the peripheral fibrous ring tissue. This is accompanied by the growth of neurovascular vessels, ultimately resulting in the relevant clinical symptoms [14]. Cellular senescence and nutritional disorders can cause severe hypoxia in the intervertebral disc region, leading to mitochondrial dysfunction that worsens intracellular energy metabolism disorders and oxidative stress. These factors accelerate cellular senescence and result in apoptosis, forming a positive feedback loop that exacerbates the progression of IVDD [15]. Hypoxic stress in the disc tissues of IVDD may result in a substantial increase in ROS levels, leading to mitochondrial dysfunction and DNA damage, which in turn leads to apoptosis or senescence [16]. In this study, we found that DHHC6 can significantly inhibit the aging of NP cells and found that hypoxia-induced apoptosis in NP cells, increased intracellular calcium and ROS levels, and decreased mitochondrial membrane potential. Upregulation of the expression level of γ-H2AX in NP cells induces the expression levels of apoptosis-related molecules Bax, Bcl, Cleaved Aspase8, Cleaved Caspase3, MMP3, MMP13, and reduces the expression of COL2 and AGG proteins.

Studies have shown that organelles do not exist independently of each other; instead, they form membrane contact sites (MCS) to mediate interorganellar communication and perform biological functions [17]. MCS are specialized regions where the membranes of different organelles are in close proximity (<50 nm) without fusion, which is the structural basis for the formation of mitochondria-associated endoplasmic reticulum membranes (MAMs) [18]. These MAMs are enriched with many organelle membrane proteins, and their localization determines the interaction between mitochondria and the endoplasmic reticulum. MAMs act as communication bridges between mitochondria and the endoplasmic reticulum. Disruption of MAM integrity can lead to mitochondrial dysfunction and endoplasmic reticulum stress, promoting pathological and physiological processes such as apoptosis and senescence. Cellular senescence promotes tissue remodeling after development and injury in the organism. However, when the accumulation of senescent cells exceeds a certain threshold, it can lead to tissue functional decline. Cellular senescence is closely related to mitochondrial function homeostasis, which is tightly regulated by calcium ions [19,20]. The transfer of Ca^2+^ to mitochondria is crucial for maintaining cellular energy and metabolic homeostasis [21]. Abnormal expression of MAMs-related proteins, changes in the structure of MAMs, and disruption of their integrity have been observed in aging diseases, including neurodegenerative diseases and myocardium [22]. Abnormal mitochondrial Ca^2+^ uptake mediated by MAMs may contribute to cellular senescence. The results of this study indicate that hypoxia induces aging of NP cells, leading to significant downregulation of functional proteins such as GRP75, VDAC1, Drp1, and Mfn2 in the MAM structure. The concomitant downregulation of the fission mediator Drp1 and the fusion protein Mfn2 suggests a coordinated suppression of mitochondrial dynamics, rather than a simple shift in the fission/fusion balance. This pattern, observed in other systems under severe stress [23,24],‌‌ likely reflects a profound dysfunction of the mitochondrial network stemming from upstream MAMs disruption. The loss of both fission and fusion capacity compromises mitochondrial quality control, leading to the accumulation of damaged organelles and contributing decisively to the senescence phenotype we observed.

The inositol 1,4,5-triphosphate receptor (IP3R) is a protein located in the endoplasmic reticulum membrane that regulates Ca^2+^ signaling from the endoplasmic reticulum to the mitochondria. It acts as a chemically gated calcium release channel by activating the inositol triphosphate (IP3) signaling cascade reaction, resulting in calcium ion delivery [23,24].‌‌ The glucose-regulated protein 75 (Grp75) acts as a bridging molecule between IP3R and voltage-dependent anion channels (VDAC). As a result, Ca^2+^ is transferred from the mitochondrial membrane gap to the mitochondrial matrix to perform its biological function. The IP3R-GRP75-VDAC complex is responsible for maintaining the structure and function of MAMs, which are involved in various cellular processes such as calcium homeostasis, endoplasmic reticulum stress, autophagy, and mitochondrial dynamics [25,26]. Studies have demonstrated that inhibiting the interaction between GRP75 and IP3R disrupts MAMs structure and communication, resulting in reduced Ca^2+^ flux [27].

S-palmitoylation is a post-translational modification (PTM) that involves attaching palmitate to cysteine residues of proteins via reversible thioester bonds. This modification plays a crucial role in regulating protein stability, subcellular localization, membrane trafficking, and interaction with effector proteins [28,29]. S-palmitoylation is a lipid modification that facilitates the migration of proteins into cellular and organelle membranes. Zhang et al. found that the palmitoylation-depalmitoylation cycle of STAT3 promotes the intracellular activation and shuttling of STAT3, leading to Th17 cells’ differentiation [30]. Fredericks et al. demonstrated that DHHC6 knockdown decreased IP3R expression in immune T cells and impaired cellular IP3R-dependent Ca^2+^ fluxes [31]. The disruption of S-palmitoylation is associated with the development of neurological, immunological, and oncological disorders and is involved in regulating cellular oxidative stress and senescence processes [32–35].

The study results indicated that hypoxia decreased the level of IP3R S-palmitoylation modification. Additionally, overexpression of DHHC6 promoted IP3R S-palmitoylation. Neither hypoxia-induced nor overexpression of DHHC6 treatment affected the level of IP3R protein expression in NP cells. However, DHHC6 overexpression significantly up-regulated the expression of functional proteins GRP75, VDAC1, Drp1, and Mfn2 in the MAMs. Furthermore, we found that DHHC6 overexpression significantly inhibited hypoxia-induced activation of ER stress markers (GRP78, CHOP, XBP1s) in NP cells. ER stress is a well-established driver of NP cell senescence and IVDD progression, and its activation is tightly coupled to MAM structural disruption and ER calcium homeostasis imbalance. Our findings suggest that DHHC6-mediated IP3R palmitoylation stabilizes MAM structure and function, thereby alleviating ER stress and subsequent NP cell senescence under hypoxic conditions. Despite revealing the role of S-palmitoylation in regulating the structural and functional abnormalities of MAMs on NP cell aging, this study still faces limitations. Firstly, despite effectively controlling experimental conditions, in vitro cell models cannot fully simulate the complex physiological and pathological environment in vivo. Secondly, this work focuses on the effect of S-palmitoylation on MAMs, neglecting the role of other modifications or signaling pathways in IVDD. Future research needs to validate the role of IP3R S-palmitoylation in animal models and comprehensively reveal the molecular mechanism of IVDD through multi-omics analysis.

## 5. Conclusions

In short, this work found that hypoxia-induced IP3R depalmitoylation mediates structural and functional abnormalities in MAMs. These abnormalities may contribute to the imbalance of cellular mitochondrial homeostasis, ER stress activation, thereby causing the NP cell senescence. This study provides an alternative for revealing the pathological mechanism of IVDD and exploring new targets for IVDD treatment.

## Supporting information

S1 Fig Schematic diagram of MAM separation.
(JPG)

S2 Fig Validation of MAM-related proteins by Western blotting.
(JPG)

S3 Fig Transmission electron microscopy (TEM) observation and quantitative analysis of the contact distance between mitochondria and endoplasmic reticulum.
(a) Box plot for quantitative statistics of the distance between endoplasmic reticulum (ER) and mitochondria in each group (unit: nm). *** indicates an extremely significant statistical difference between groups (P < 0.001); (b) Representative TEM images of cells in each group. The lower panels are magnified views of the dotted box area in the corresponding upper panels, showing the ultrastructure of mitochondria (M), endoplasmic reticulum (ER) and mitochondria-associated ER membrane (MAM). Scale bar = 500 nm, n = 6 biological replicates per group.
(JPG)

S4 Fig DHHC6 overexpression preserves endoplasmic reticulum (ER) Ca^2+^ homeostasis under hypoxia-ischemia (HI) injury.
(A) Dynamic changes of ER Ca^2+^ signaling in NP cells under different experimental conditions detected by Fluo-4 AM fluorescent probe (n = 6, ns: not significant, P > 0.05). (B) Quantitative analysis of ER Ca^2+^ content in NP cells after thapsigargin-mediated SERCA inhibition(n = 6, ***P < 0.001).
(JPG)

S1 File Raw images.
(PDF)

S2 File Minimal data set.
(XLSX)

S3 File TEM images.
(ZIP)

## Abbreviation

IVDD: intervertebral disc degeneration
NPC: nucleus pulposus cell
MAMs: mitochondria-associated membranes
Bax: BCL-2-associated X protein
MMP3: matrix metalloproteinase3
MMP13: matrix metalloproteinase13
COL2: collagen type II
AGG: aggrecan
IP3R: inositol 1,4,5-trisphosphate receptor
GRP75: glucose regulated protein
Drp1: dynamin-related protein 1
Mfn2: Mitofusin 2
ROS: reactive oxygen species
APT2: Lysophospholipase A2
HI: hypoxia-induced
OE-DHHC6: overexpression of DHHC6
OE-NC: overexpression of negative control
