# Supplementary figures and images for: S-palmitoylation regulates the function of the mitochondria-associated endoplasmic reticulum membrane to alleviate the senescence of nucleus pulposus cells
Source: PLoS One. 2026 May 22;21(5):e0348801. doi: 10.1371/journal.pone.0348801 (PMC13196933; doi:10.1371/journal.pone.0348801)

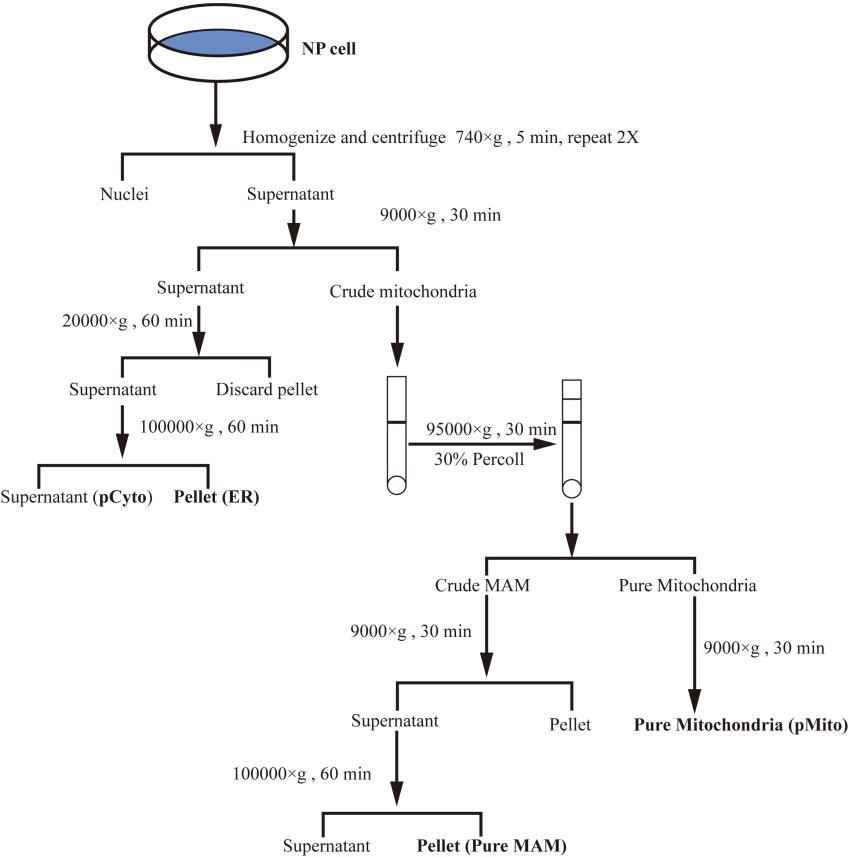

Supplement: S1 Fig — Schematic diagram of MAM separation. (JPG) [file pone.0348801.s001.jpg]

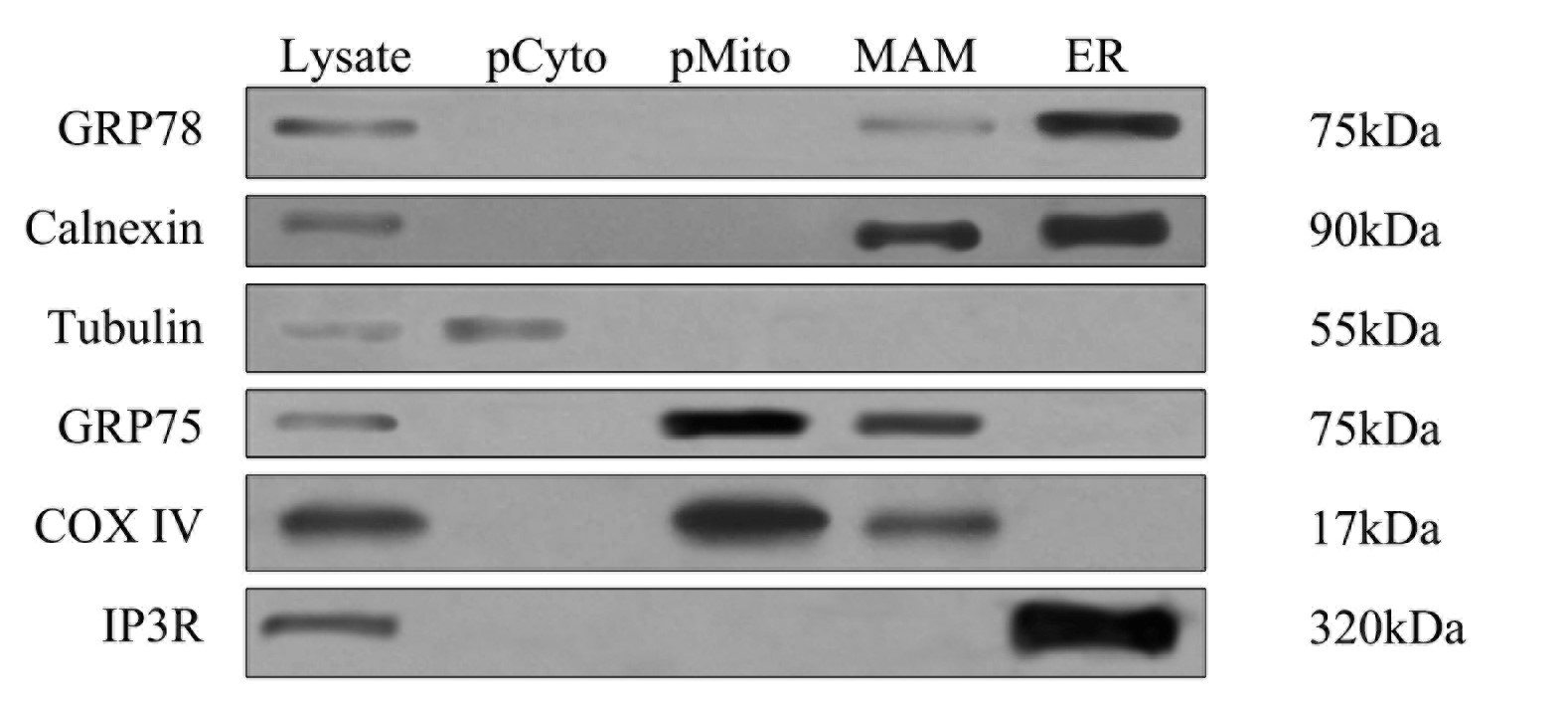

Supplement: S2 Fig — Validation of MAM-related proteins by Western blotting. (JPG) [file pone.0348801.s002.jpg]

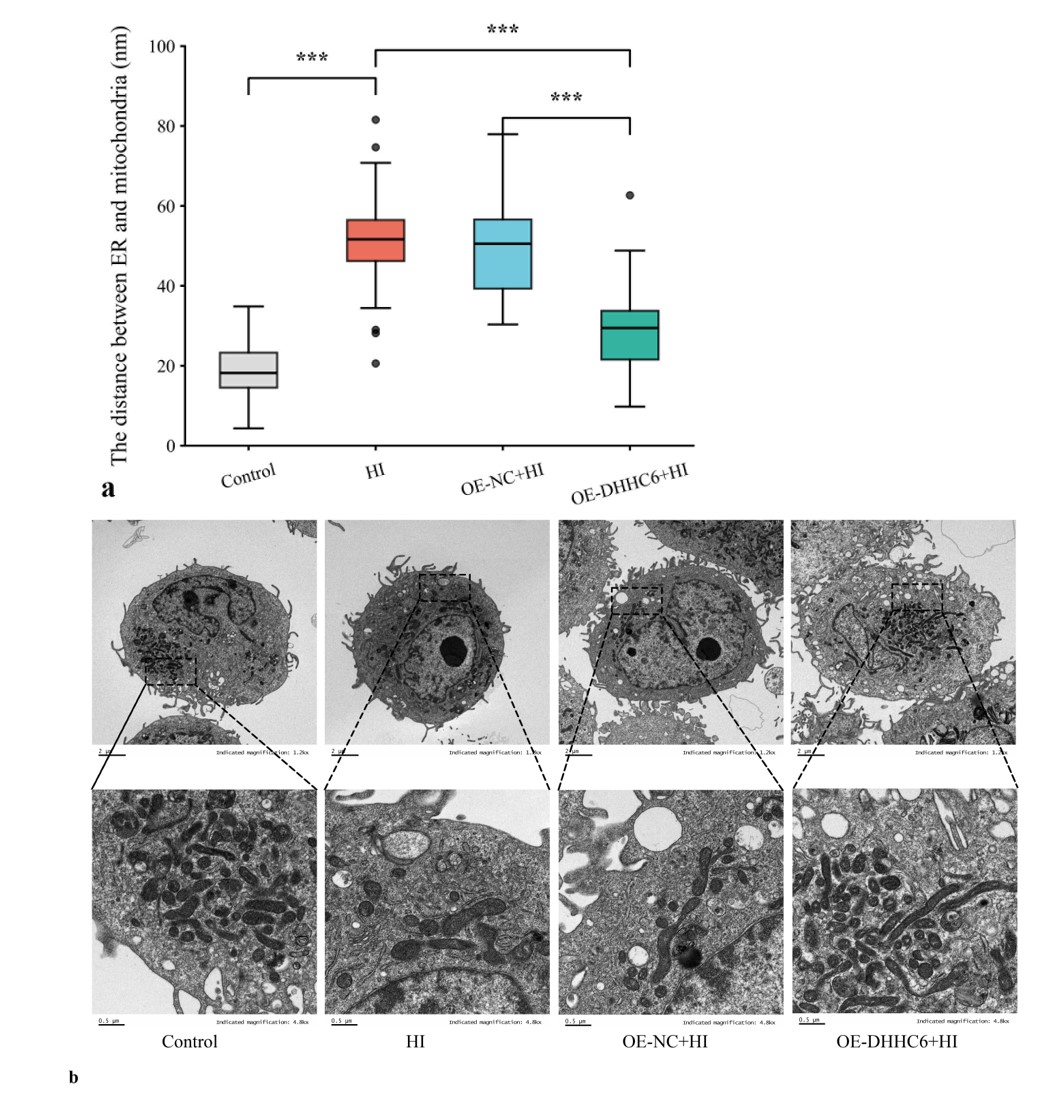

Supplement: S3 Fig — Transmission electron microscopy (TEM) observation and quantitative analysis of the contact distance between mitochondria and endoplasmic reticulum. (a) Box plot for quantitative statistics of the distance between endoplasmic reticulum (ER) and mitochondria in each group (unit: nm). *** indicates an extremely significant statistical difference between groups (P < 0.001); (b) Representative TEM images of cells in each group. The lower panels are magnified views of the dotted box area in the corresponding upper panels, showing the ultrastructure of mitochondria (M), endoplasmic reticulum (ER) and mitochondria-associated ER membrane (MAM). Scale bar = 500 nm, n = 6 biological replicates per group. (JPG) [file pone.0348801.s003.jpg]

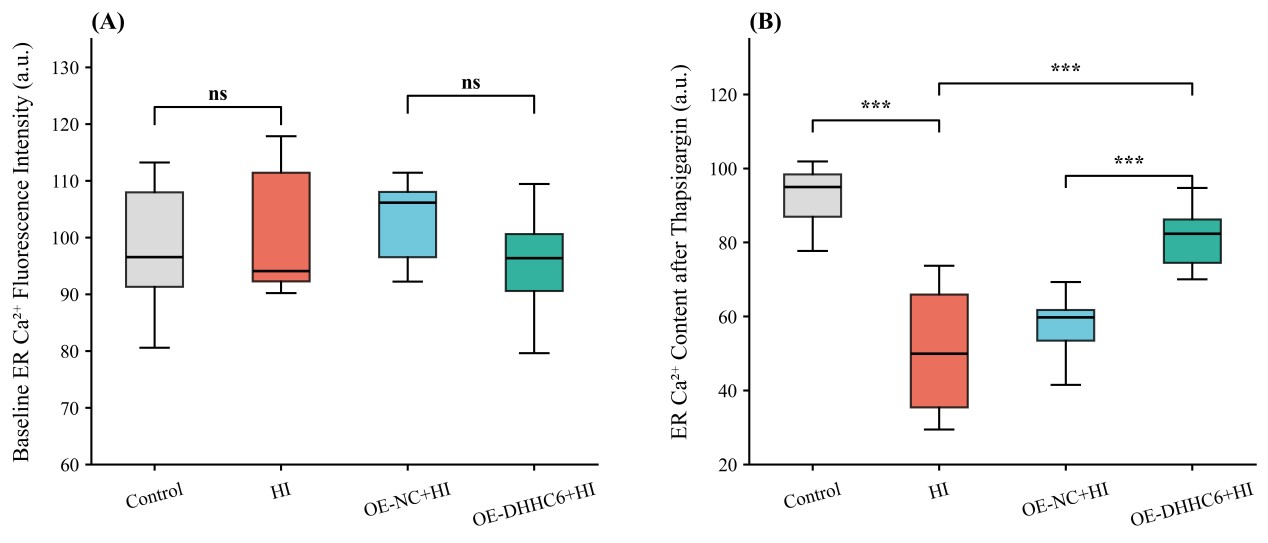

Supplement: S4 Fig — DHHC6 overexpression preserves endoplasmic reticulum (ER) Ca2+ homeostasis under hypoxia-ischemia (HI) injury. (A) Dynamic changes of ER Ca2+ signaling in NP cells under different experimental conditions detected by Fluo-4 AM fluorescent probe (n = 6, ns: not significant, P > 0.05). (B) Quantitative analysis of ER Ca2+ content in NP cells after thapsigargin-mediated SERCA inhibition(n = 6, ***P < 0.001). (JPG) [file pone.0348801.s004.jpg]

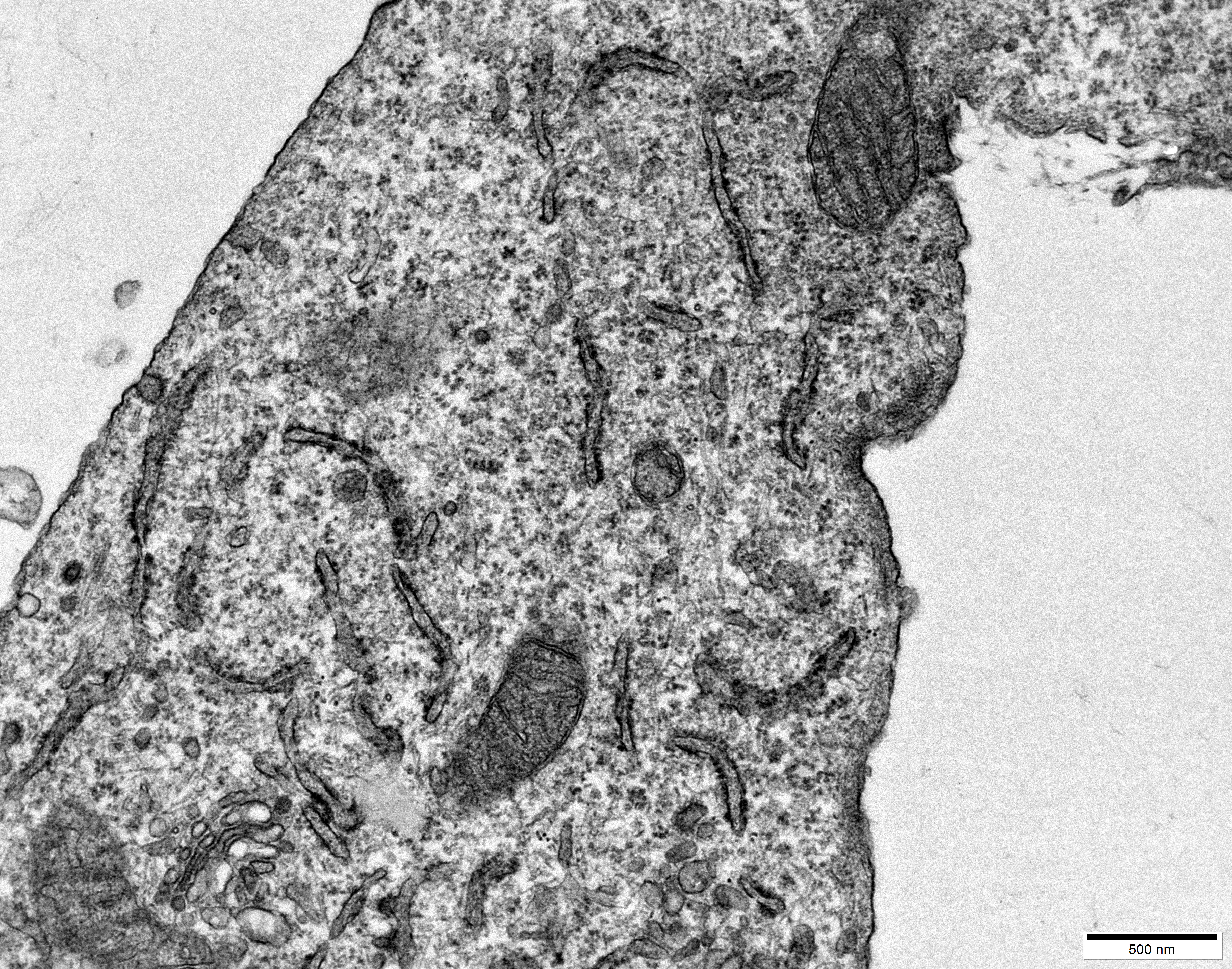

Supplement: S3 File — TEM images. (ZIP) [file pone.0348801.s007.zip › ═╕╔Σ╡τ╛╡/CTR.png]

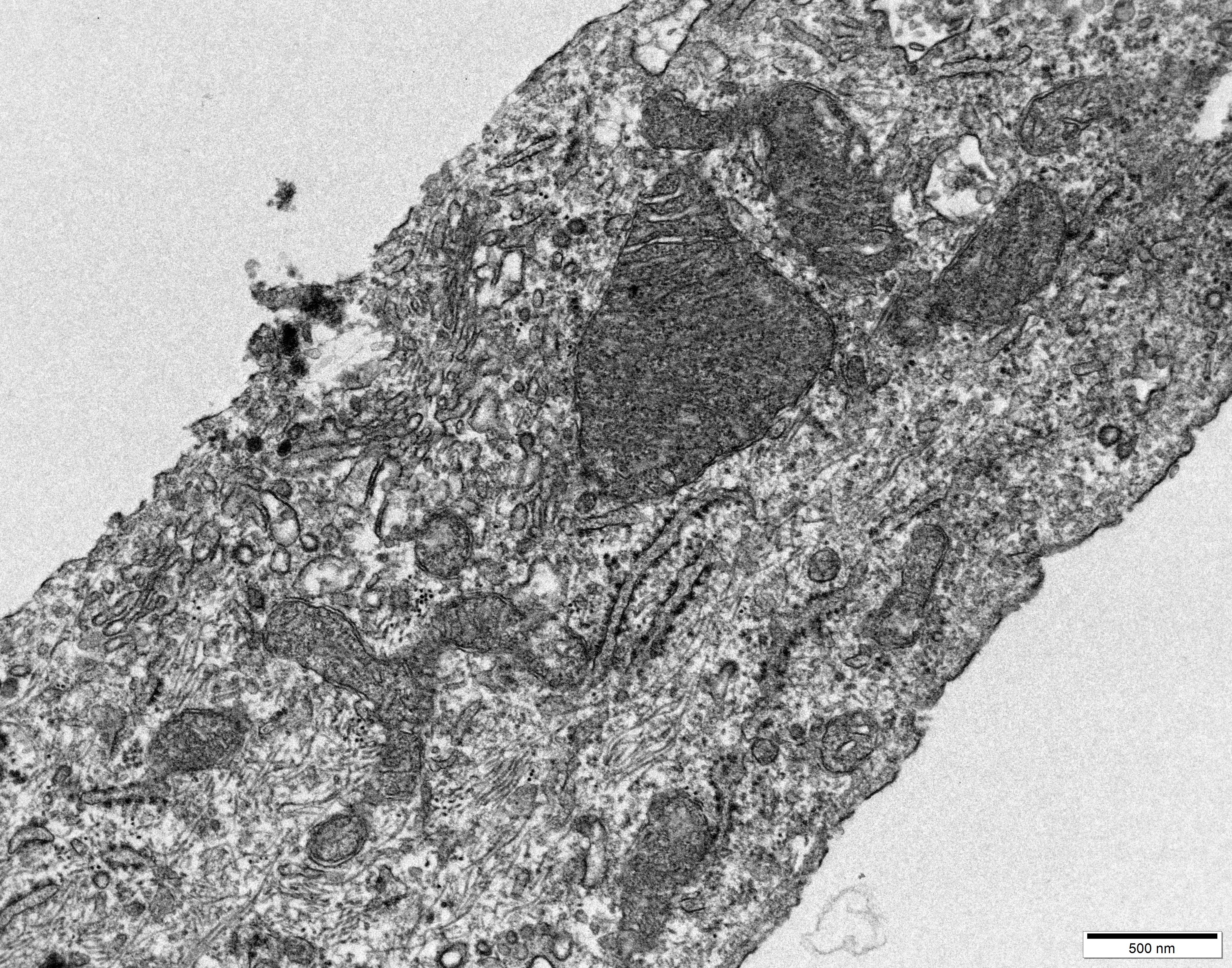

Supplement: S3 File — TEM images. (ZIP) [file pone.0348801.s007.zip › ═╕╔Σ╡τ╛╡/Hypoxia.png]

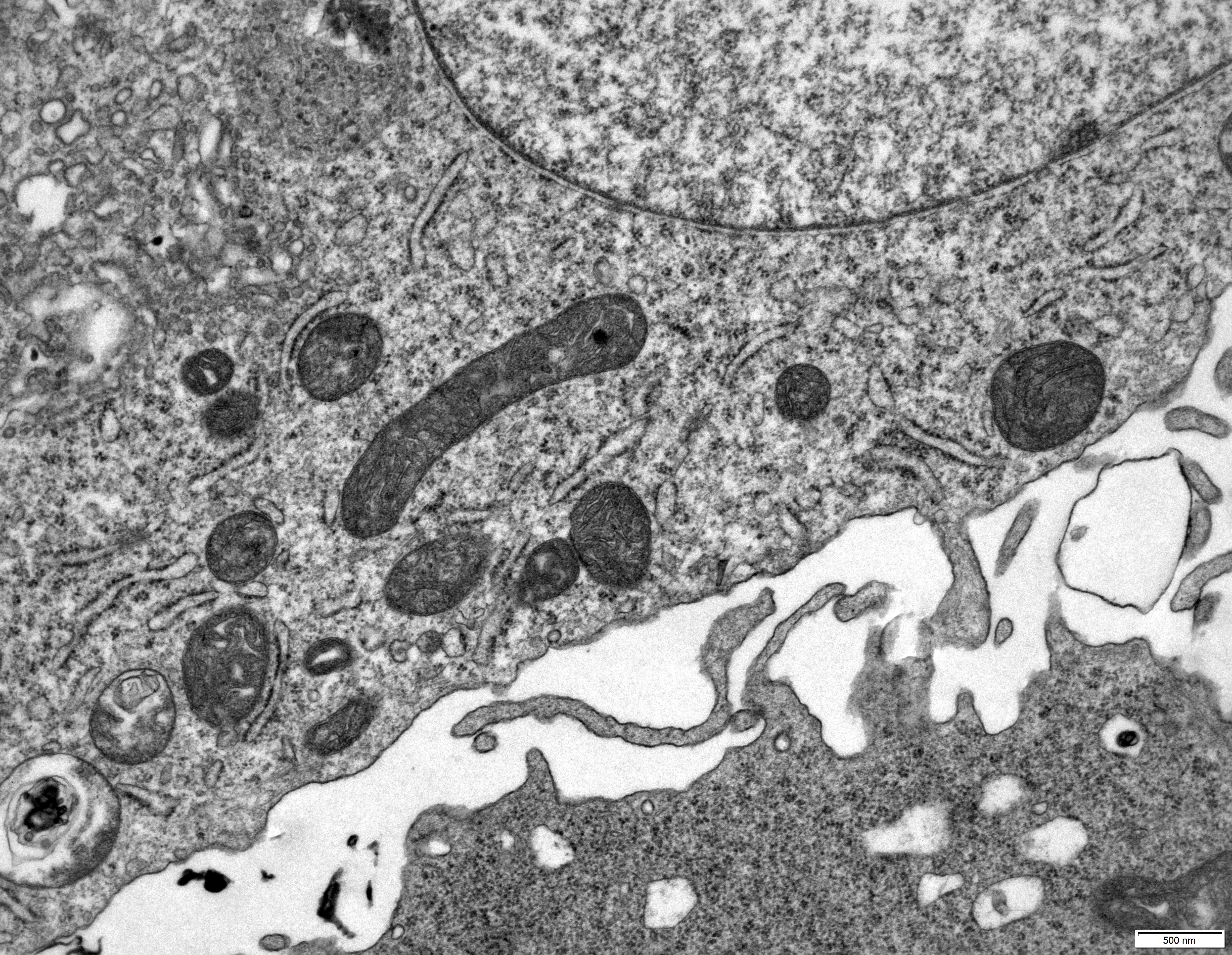

Supplement: S3 File — TEM images. (ZIP) [file pone.0348801.s007.zip › ═╕╔Σ╡τ╛╡/OE-DHHC6+HI.png]

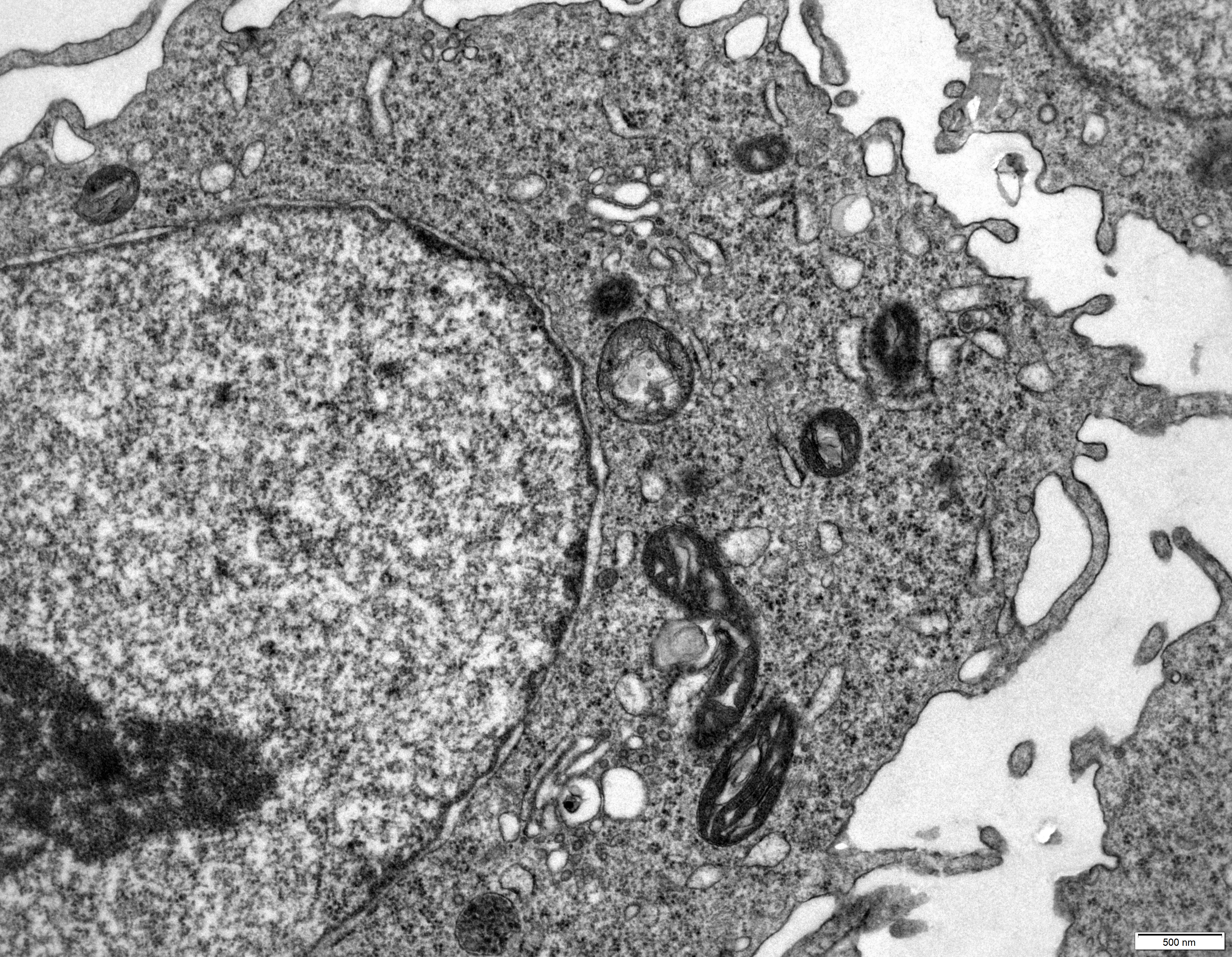

Supplement: S3 File — TEM images. (ZIP) [file pone.0348801.s007.zip › ═╕╔Σ╡τ╛╡/OE-NC+HI.png]
