# Supplementary material for: S-palmitoylation regulates the function of the mitochondria-associated endoplasmic reticulum membrane to alleviate the senescence of nucleus pulposus cells
Source: PLoS One. 2026 May 22;21(5):e0348801. doi: 10.1371/journal.pone.0348801 (PMC13196933; doi:10.1371/journal.pone.0348801)
Supplement: S1 File — Raw images. (PDF) [file pone.0348801.s005.pdf]

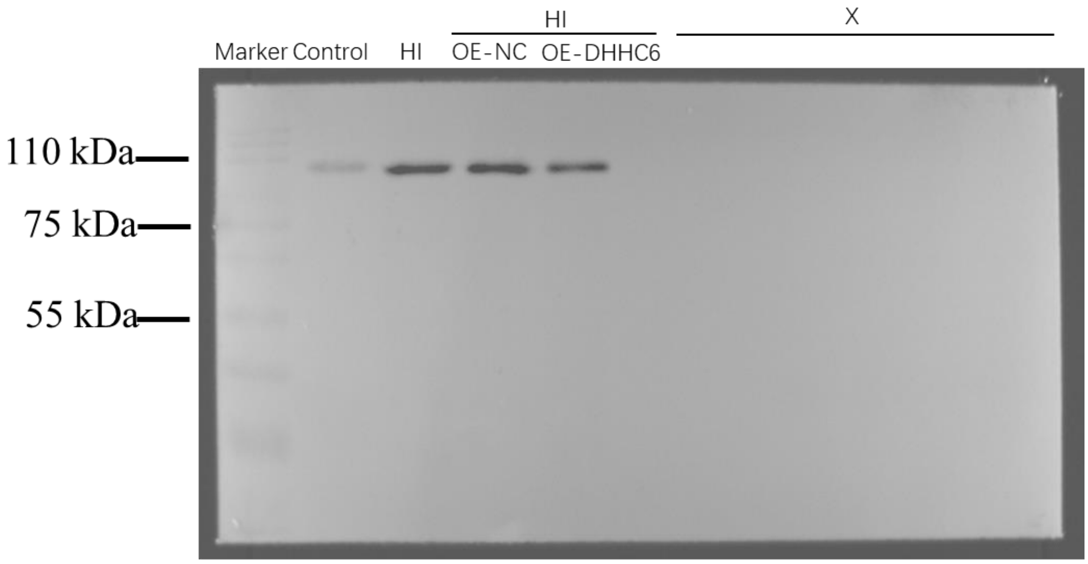

RB-105KDa

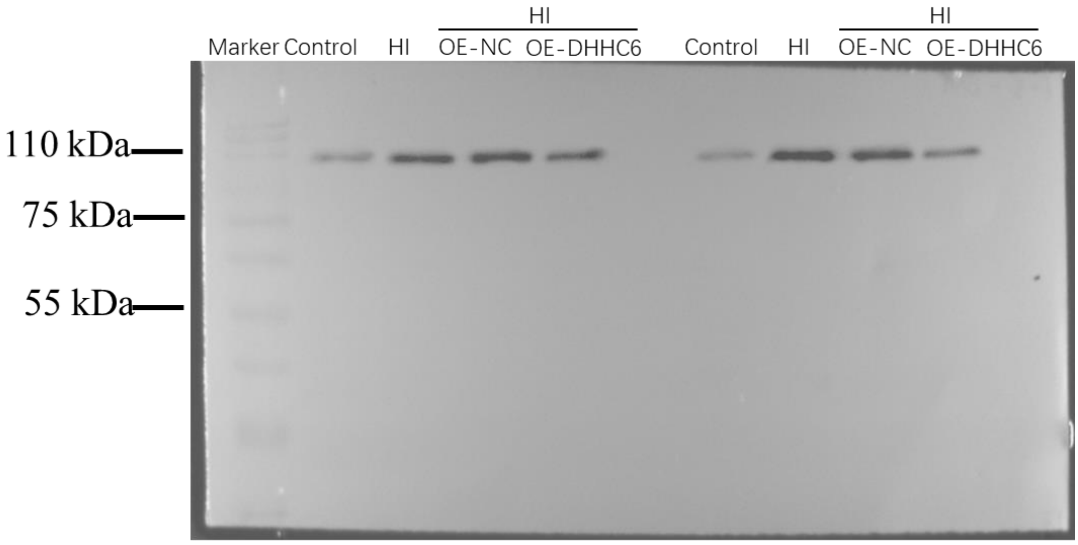

RB-105KDa

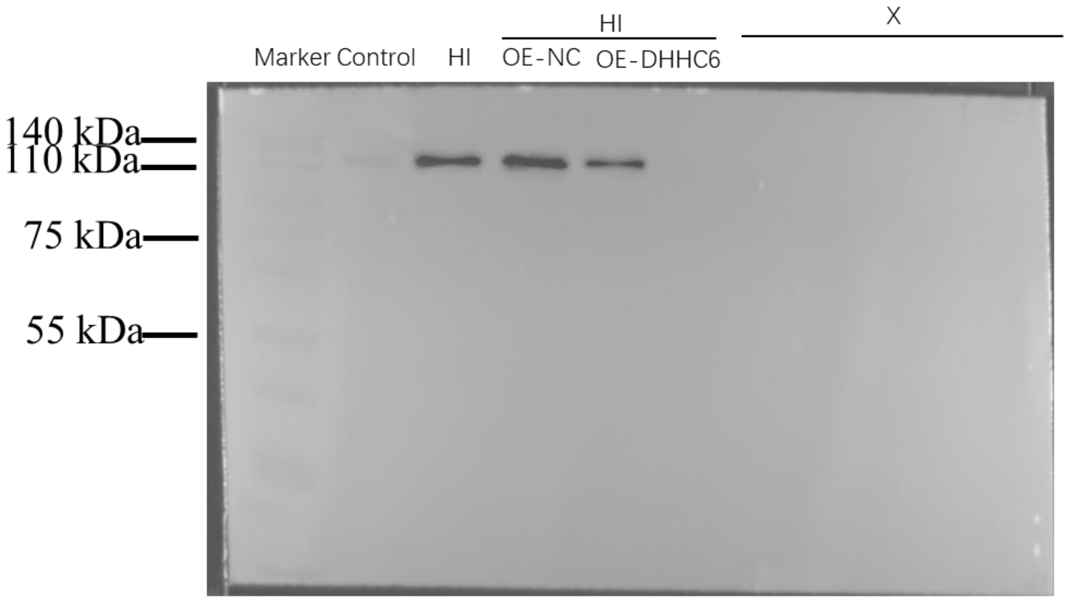

RB2-128KDa

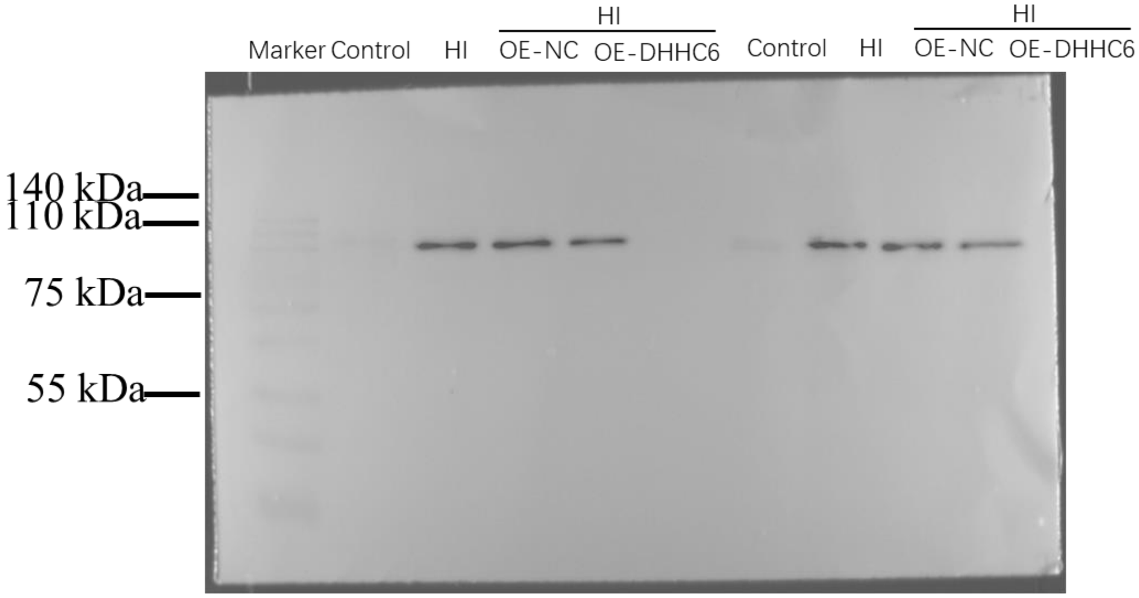

RB2-128KDa

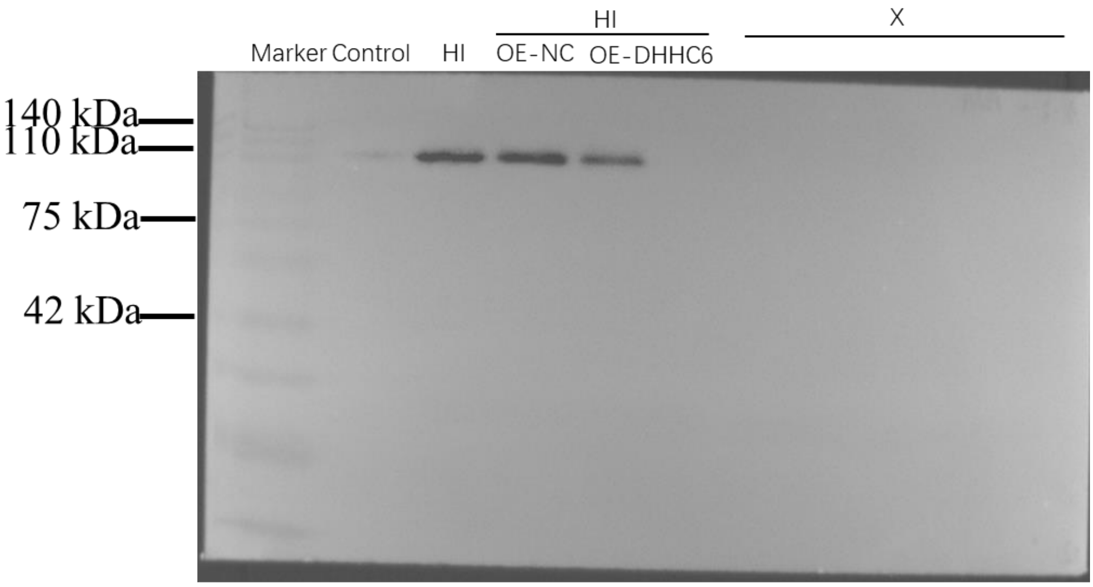

p107-121KDa

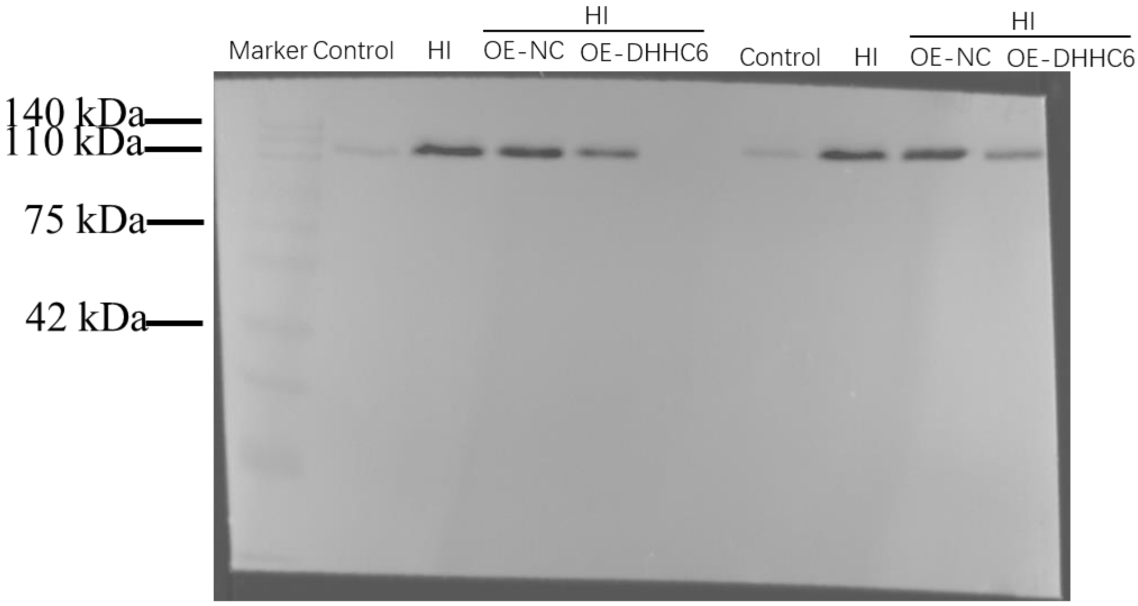

p107-121KDa

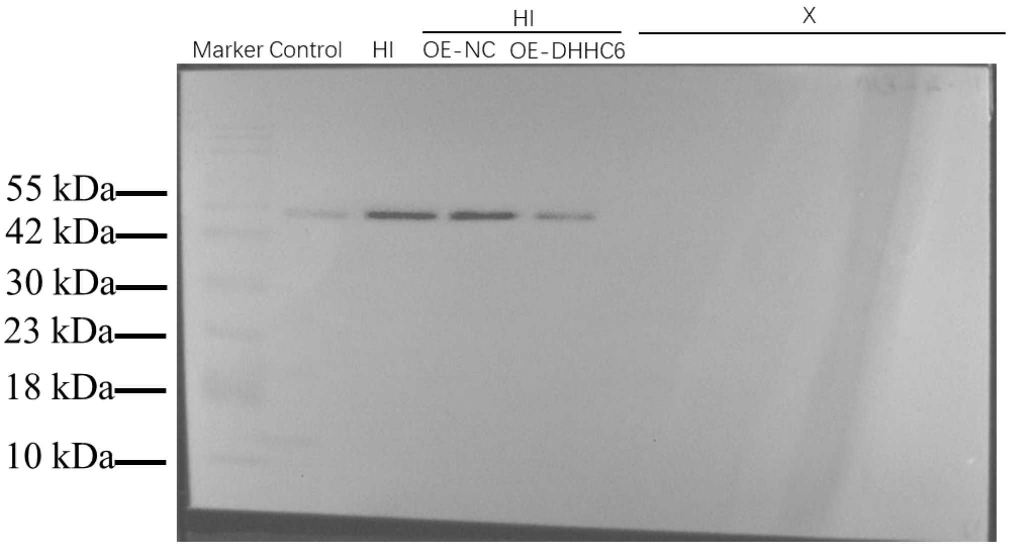

p53-53KDa

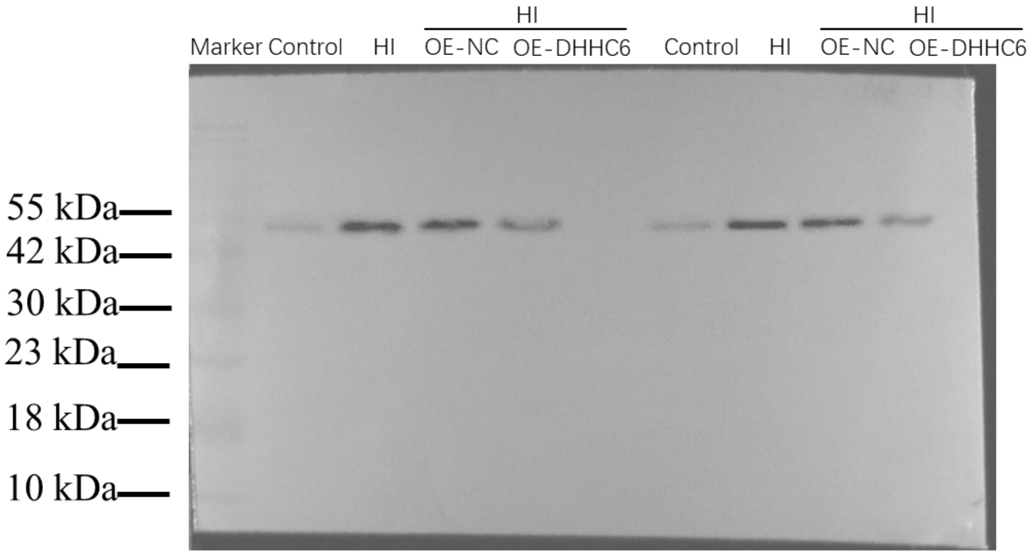

p53-53KDa

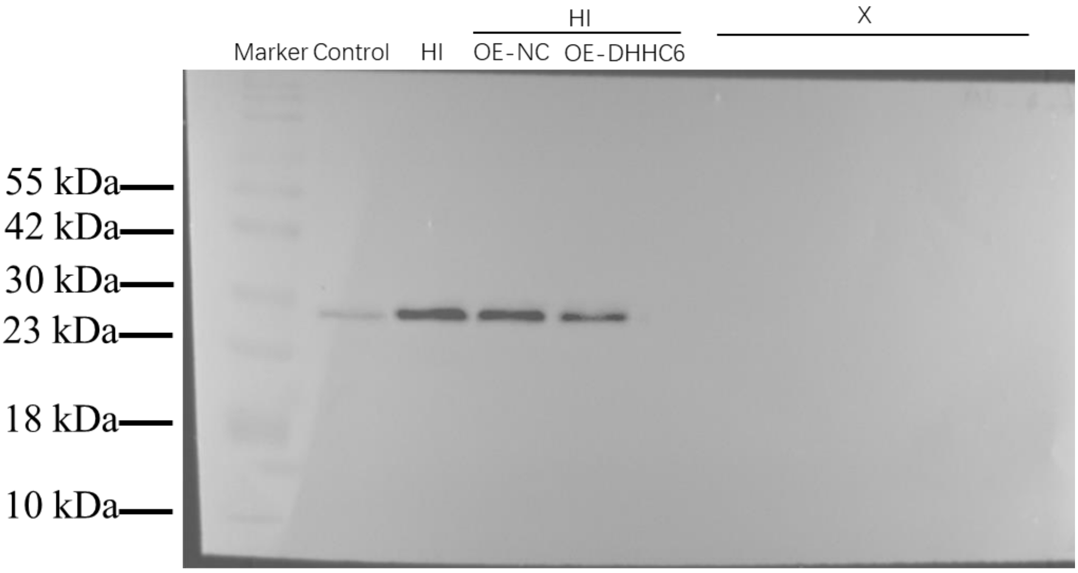

p27-27KDa

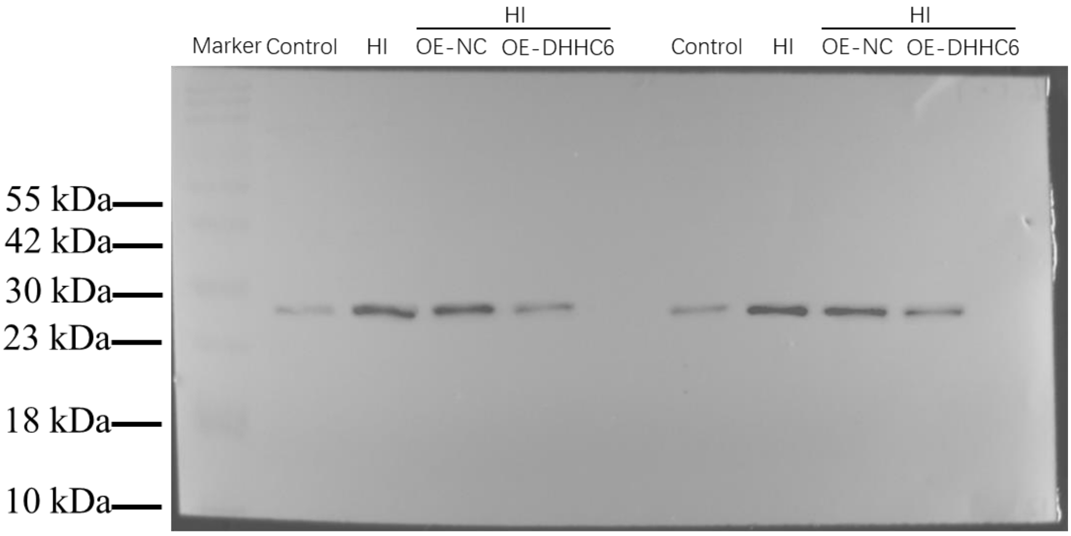

p27-27KDa

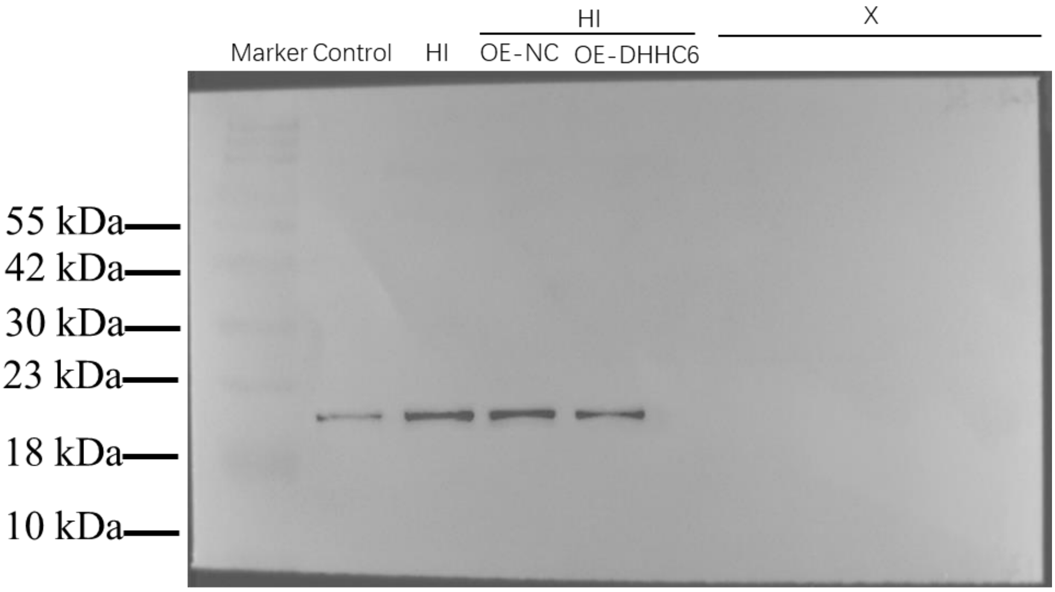

p21-21KDa

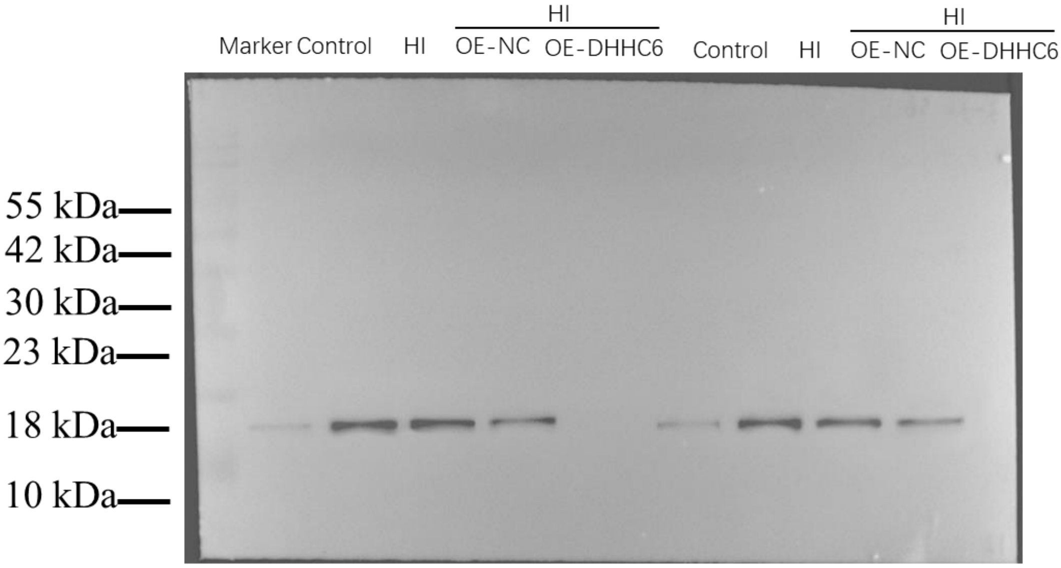

p21-21KDa

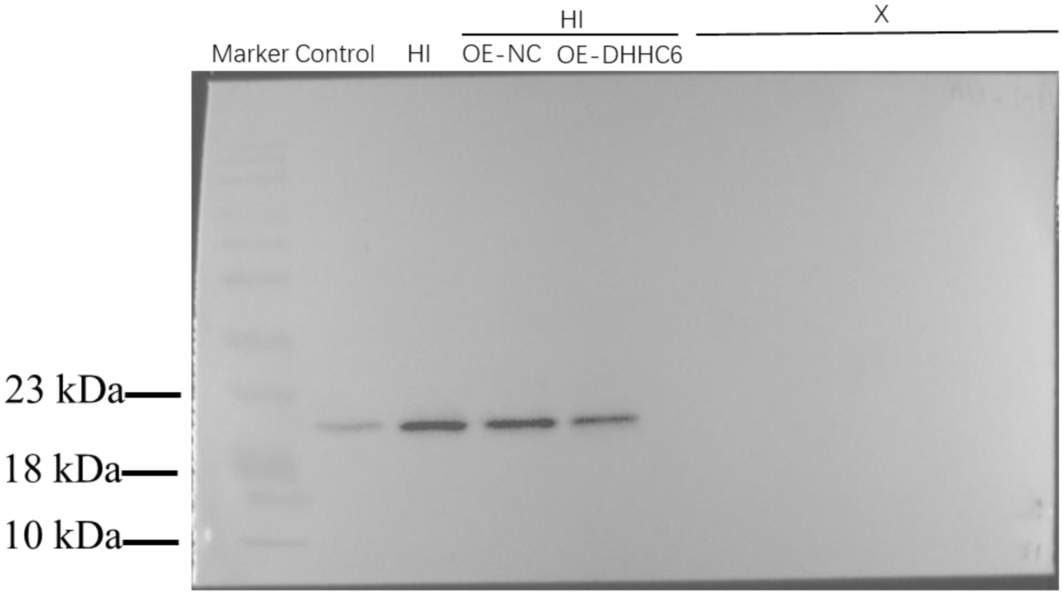

ARF-21KDa

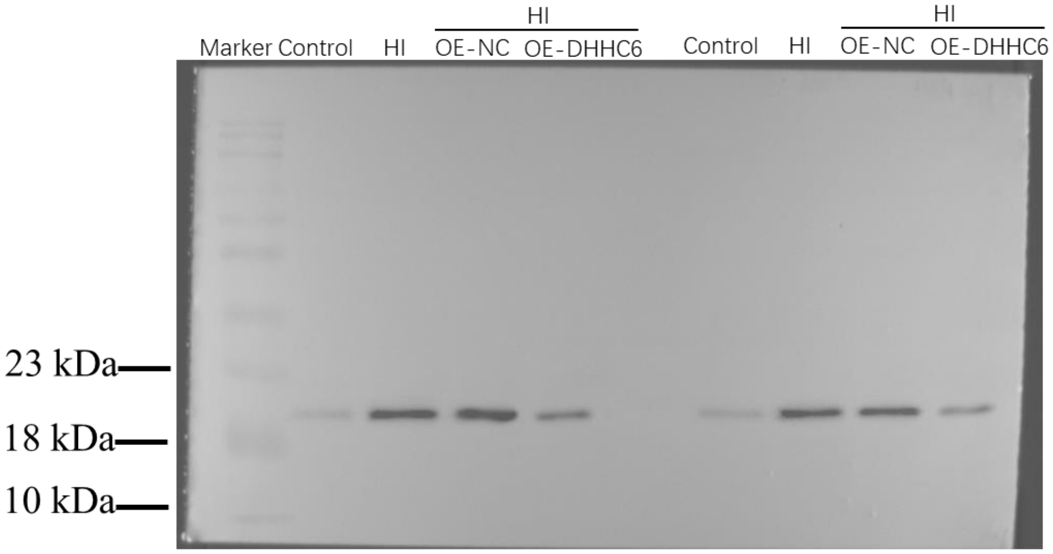

ARF-21KDa

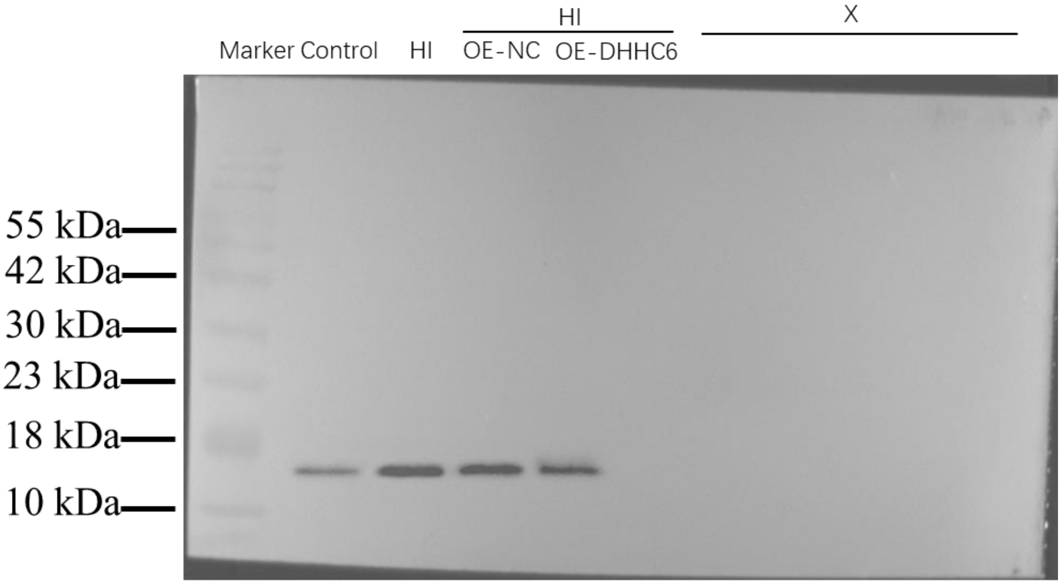

p16-16KDa

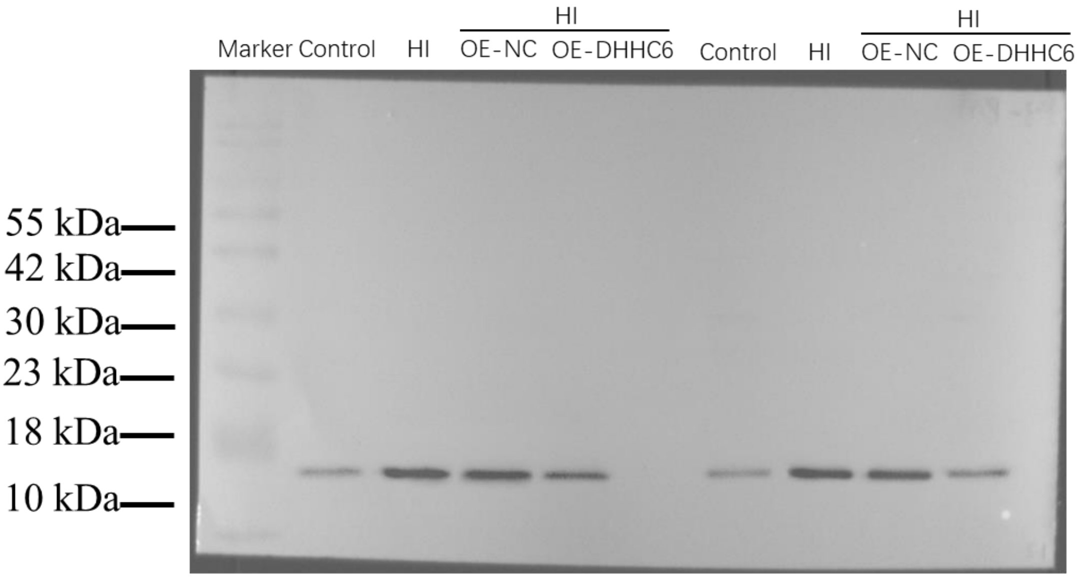

p16-16KDa

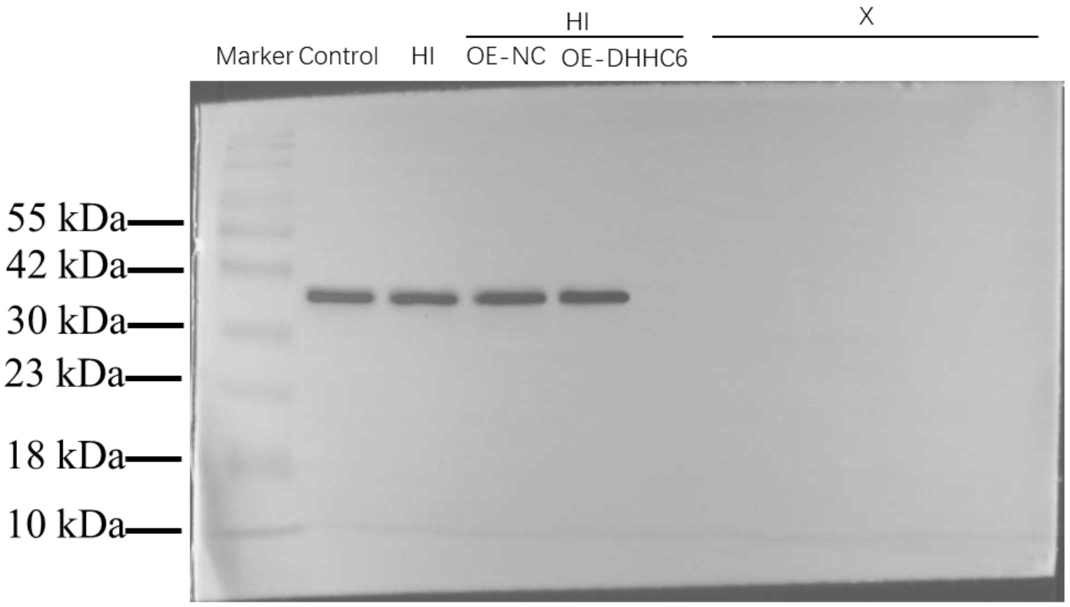
HI
X
Marker Control
HI
OE-NC
OE-DHHC6
55 kDa
42 kDa
30 kDa
23 kDa
18 kDa
10 kDa

GADPH

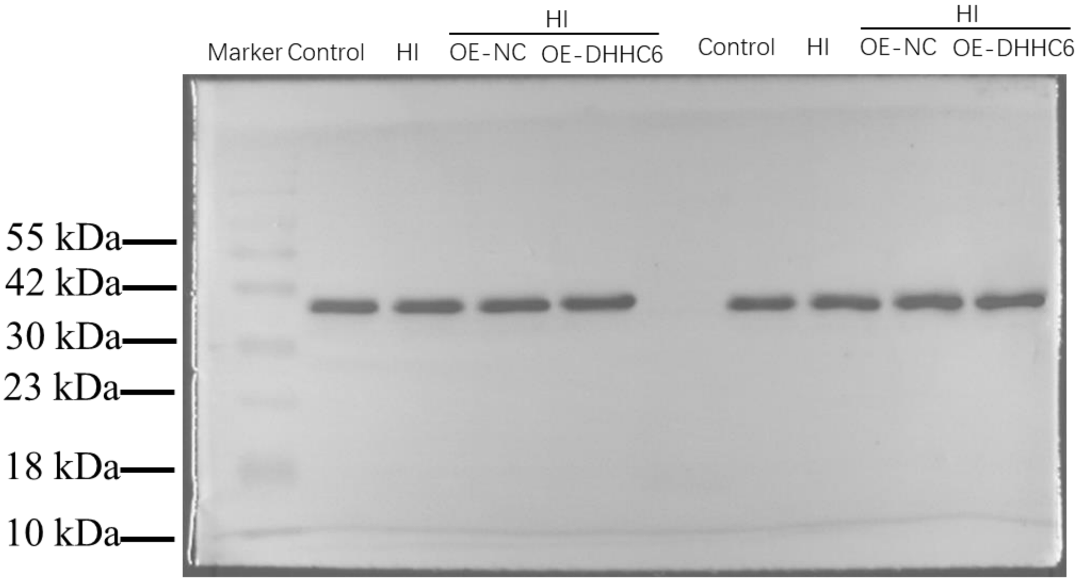
HI
HI
Marker Control
HI
OE-NC
OE-DHHC6
Control
HI
OE-NC
OE-DHHC6
55 kDa
42 kDa
30 kDa
23 kDa
18 kDa
10 kDa

GADPH

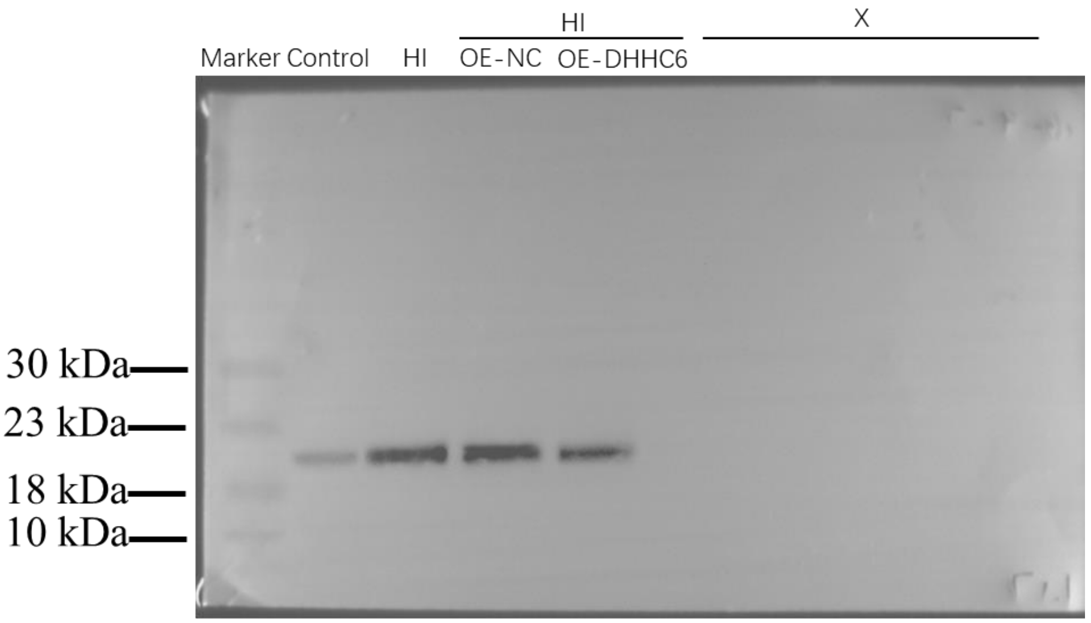

Bax -21 kDa

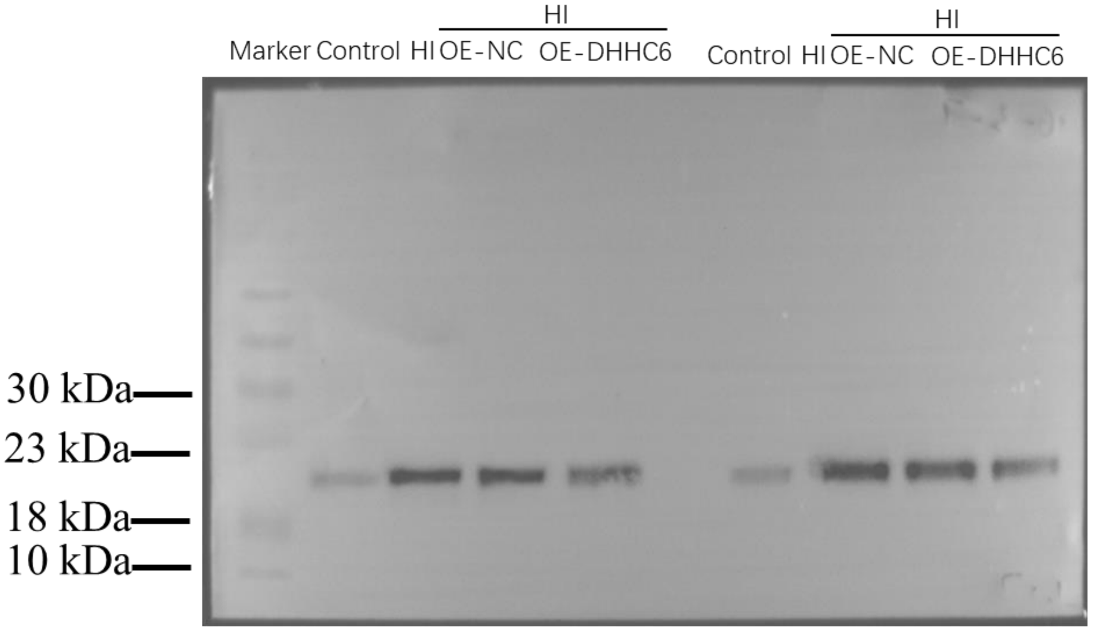

Bax -21 kDa

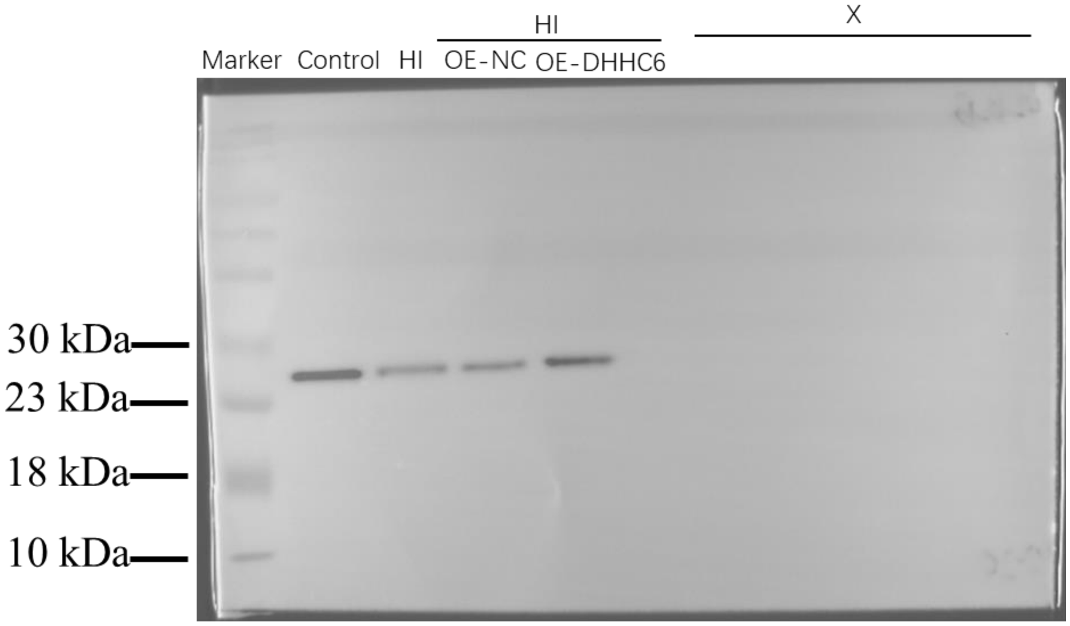

Bcl2 -26 kDa

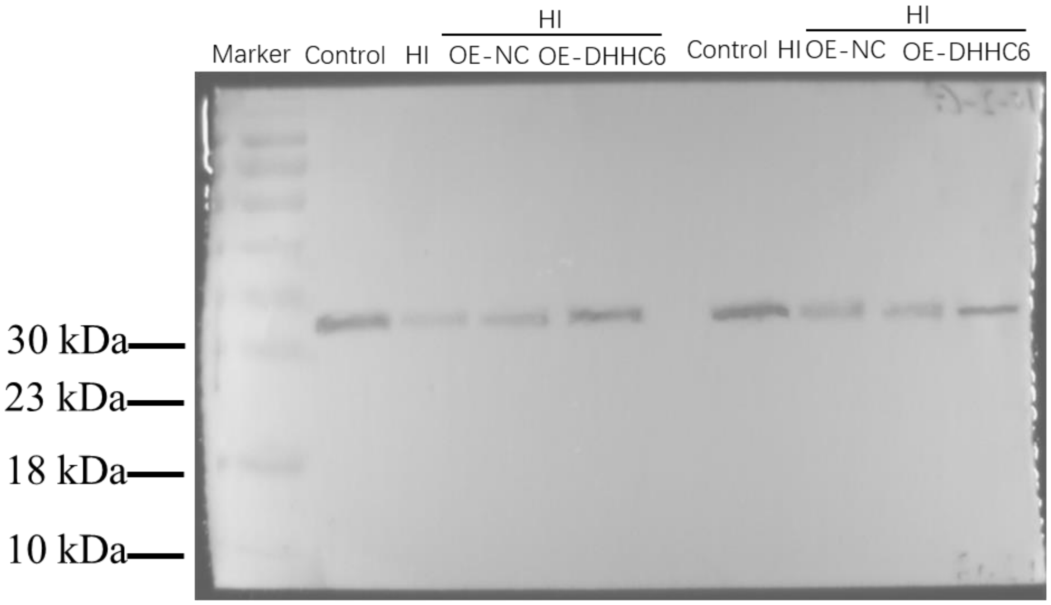

Bcl2 -26 kDa

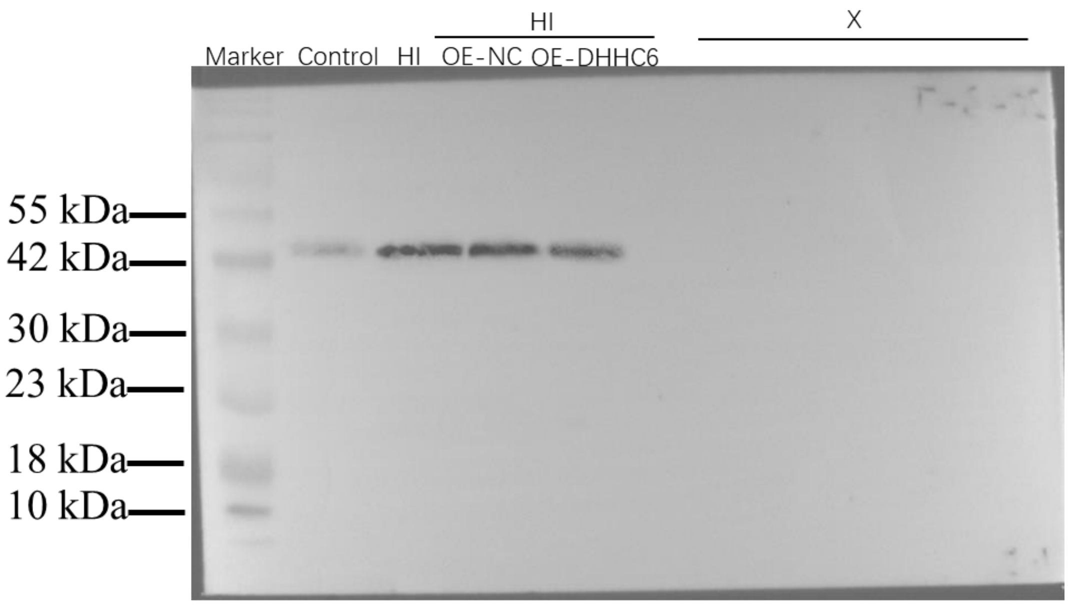

Cleaved Caspase8 -45 kDa

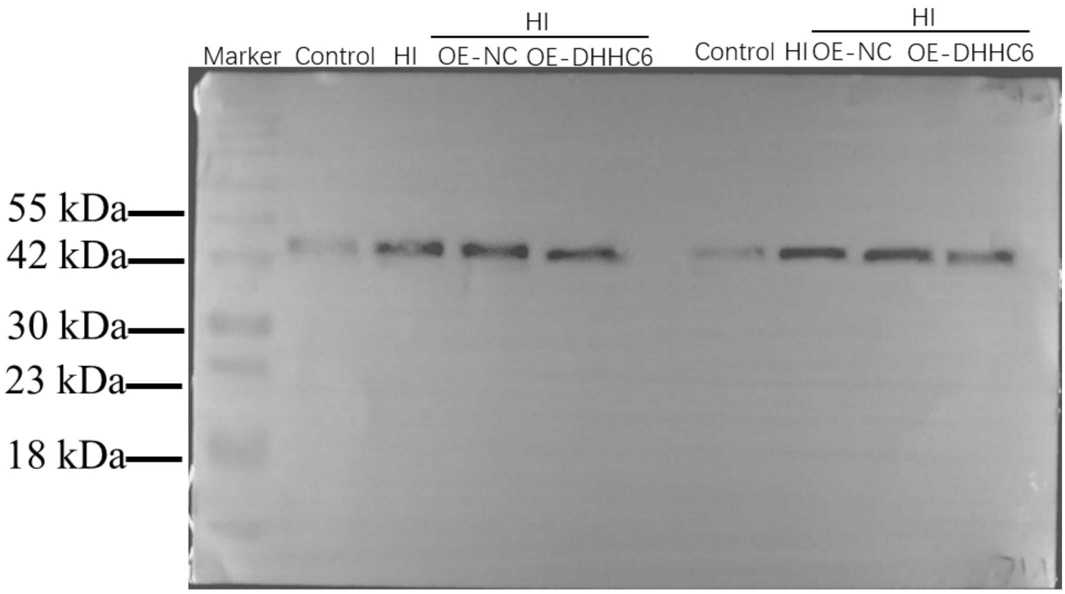

Cleaved Caspase8 -45 kDa

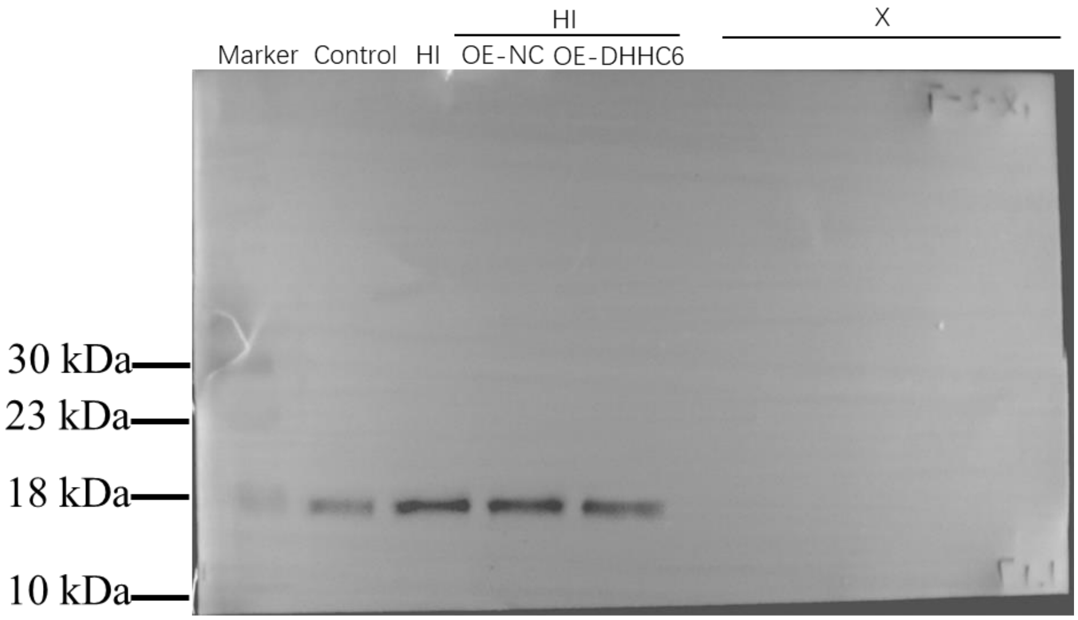

Cleaved Caspase3 -17 kDa

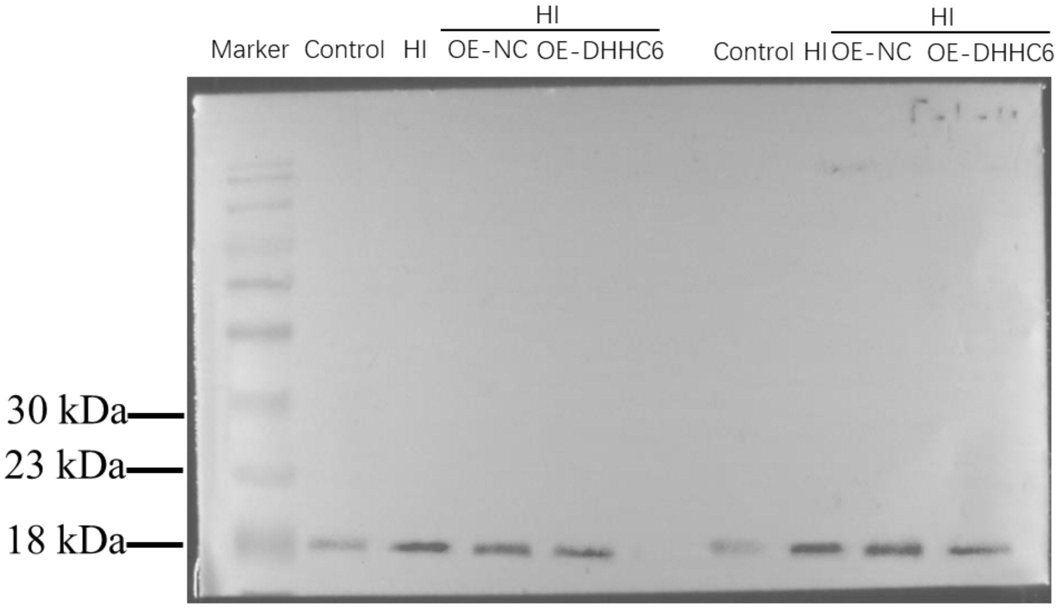

Cleaved Caspase3 -17 kDa

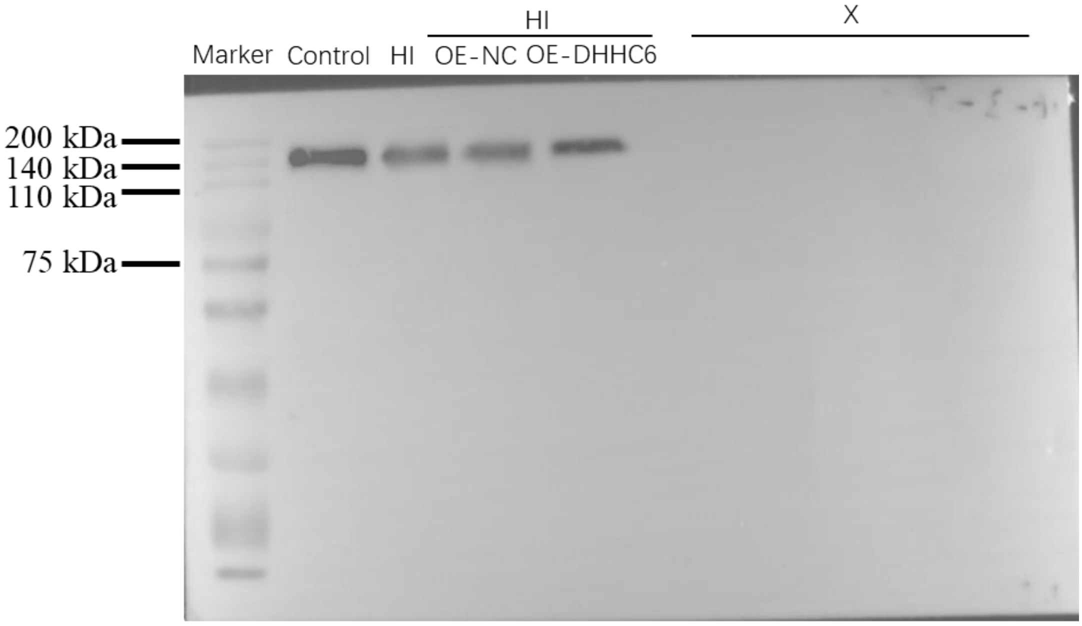

Col2 -150 kDa

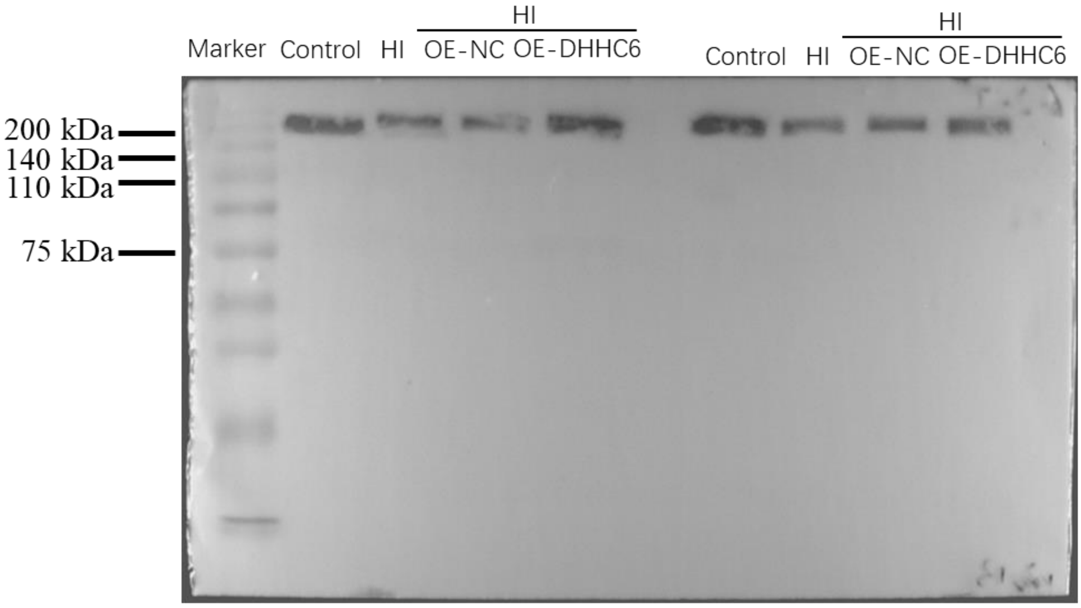

Col2 -150 kDa

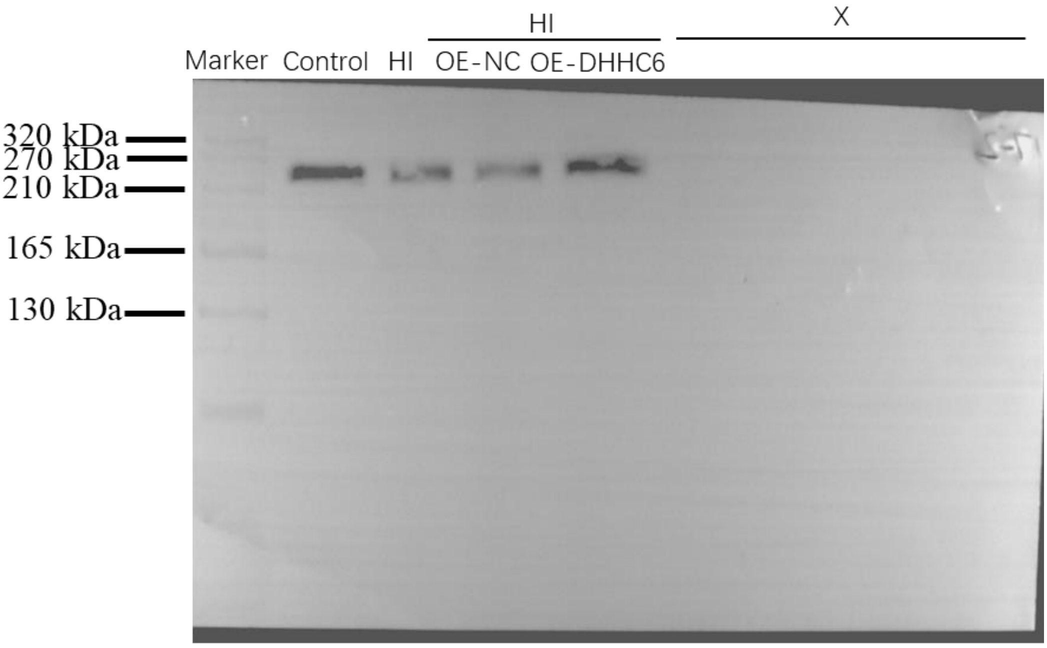

AGG-250 kDa

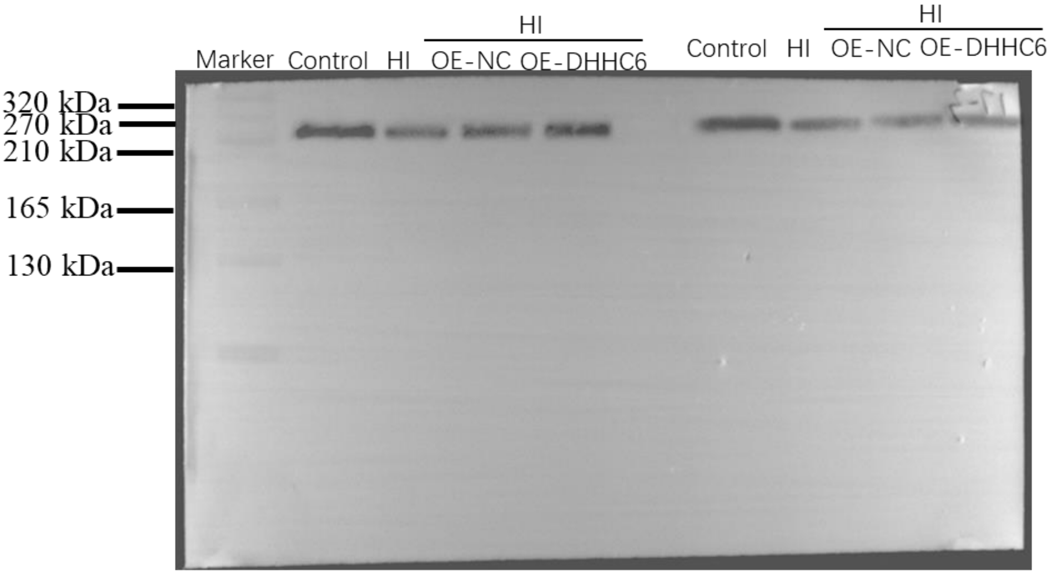

AGG-250 kDa

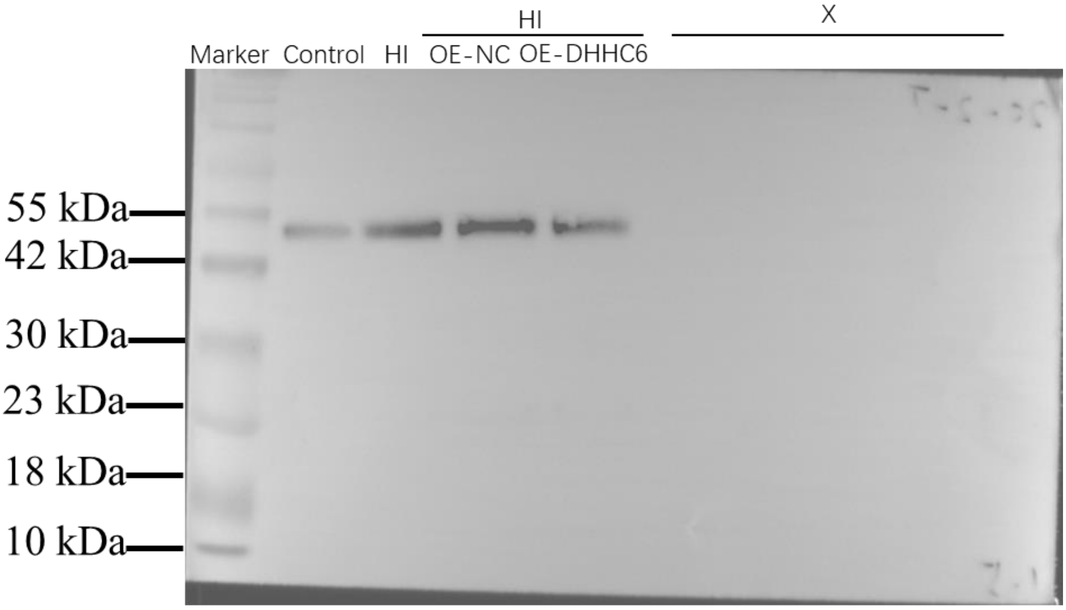

MMP3 -50 kDa

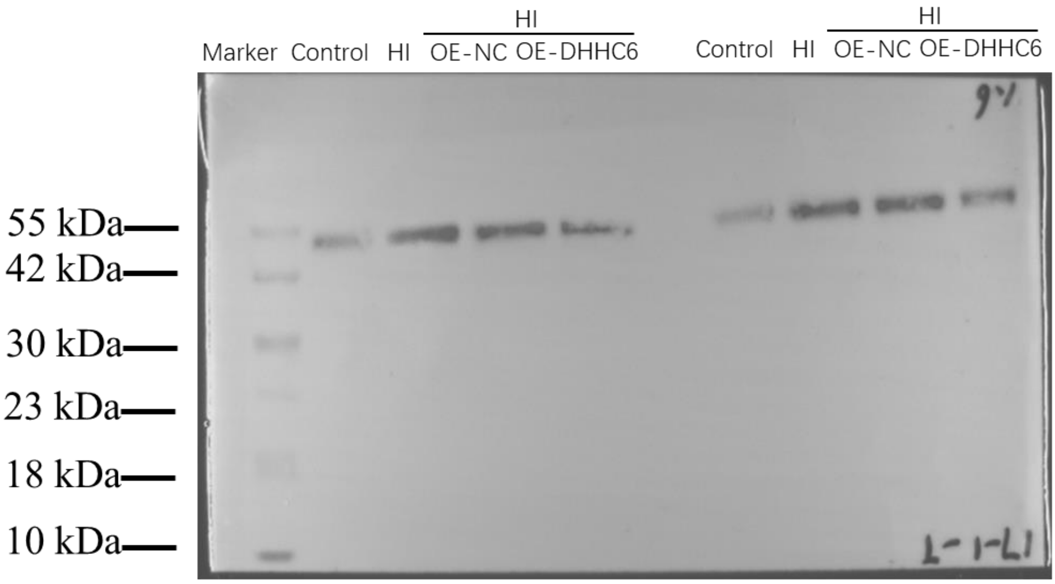

MMP3 -50 kDa

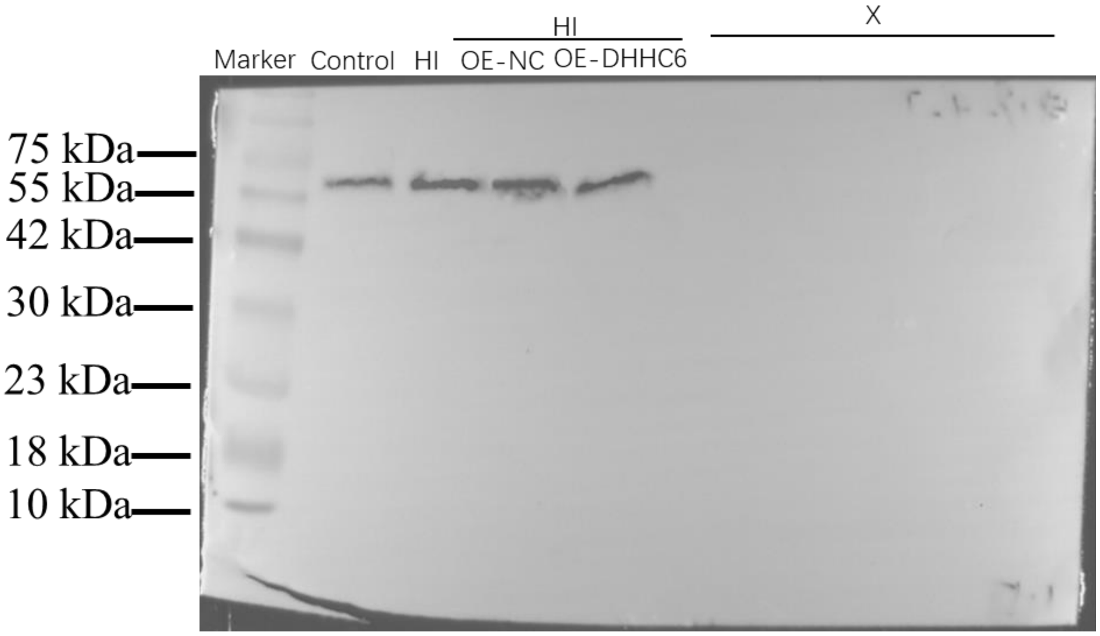

MMP13 -60 kDa

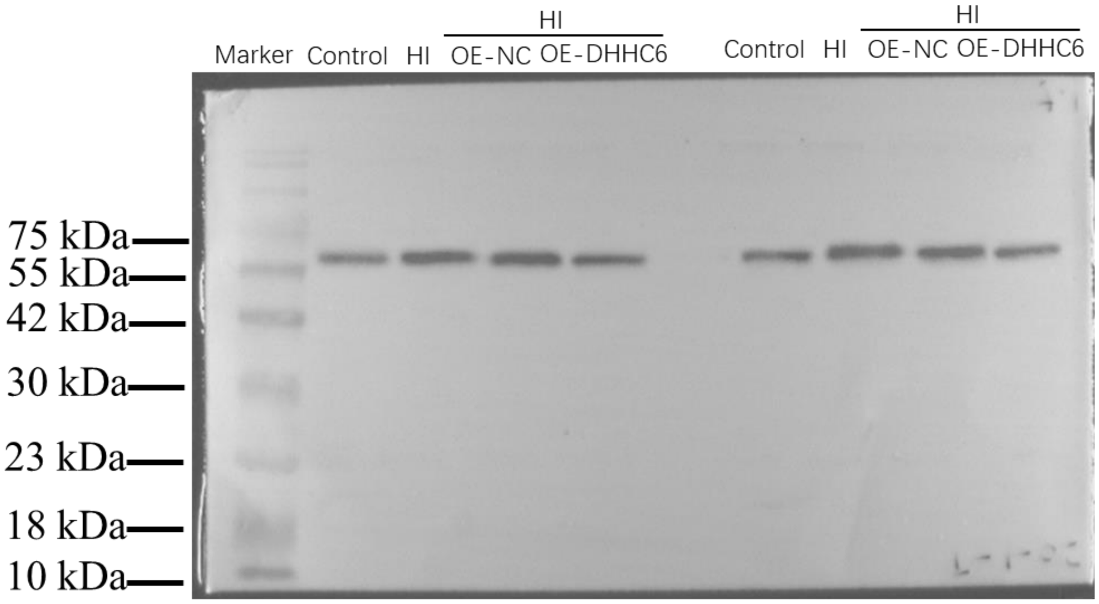

MMP13 -60 kDa

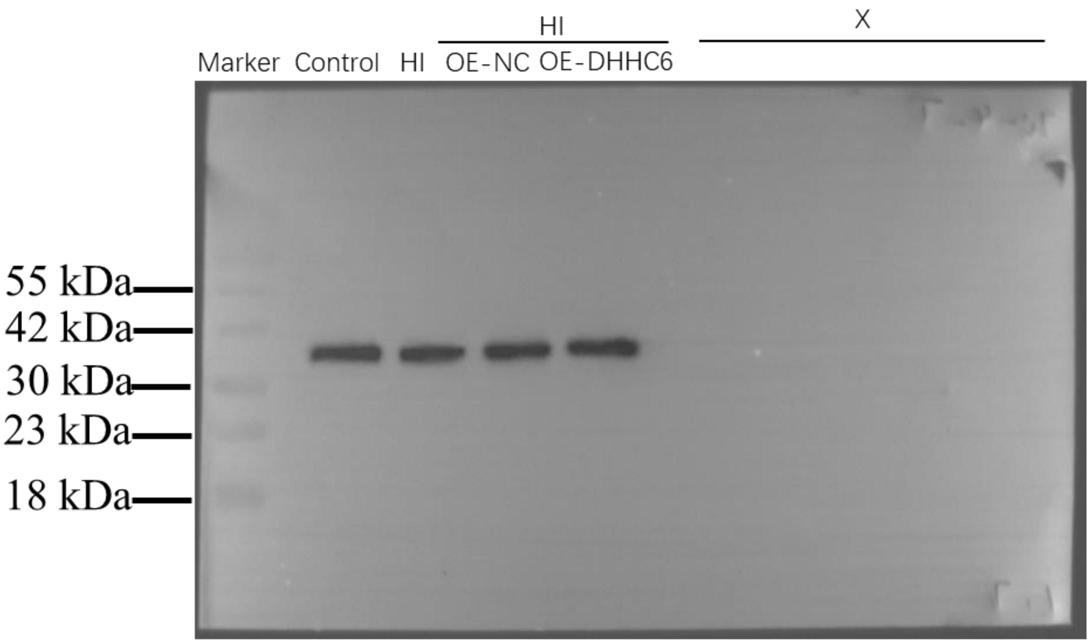

GAPDH-36 kDa

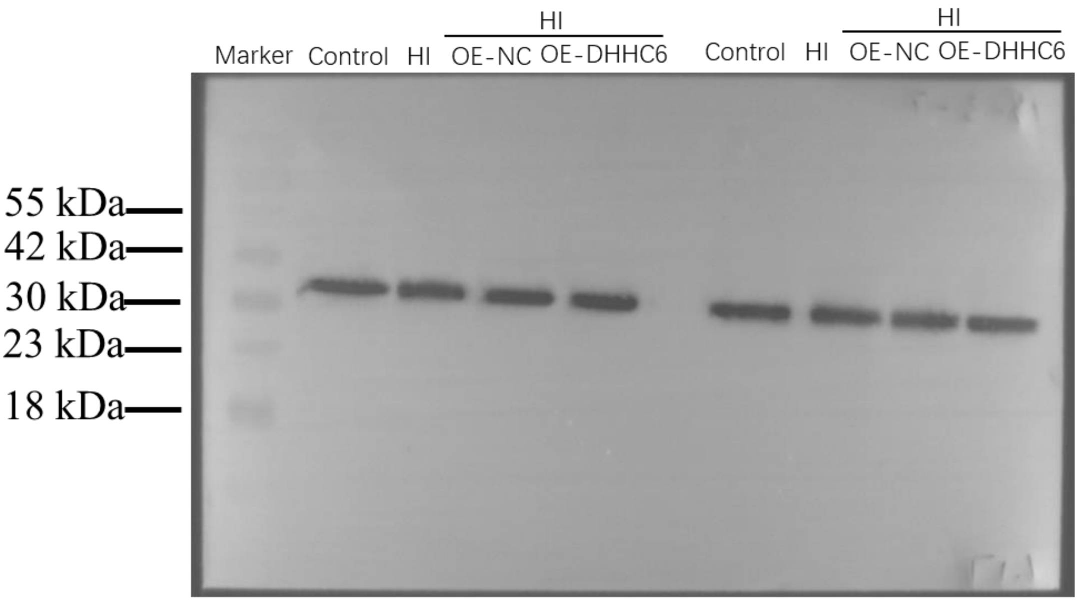

GAPDH-36 kDa

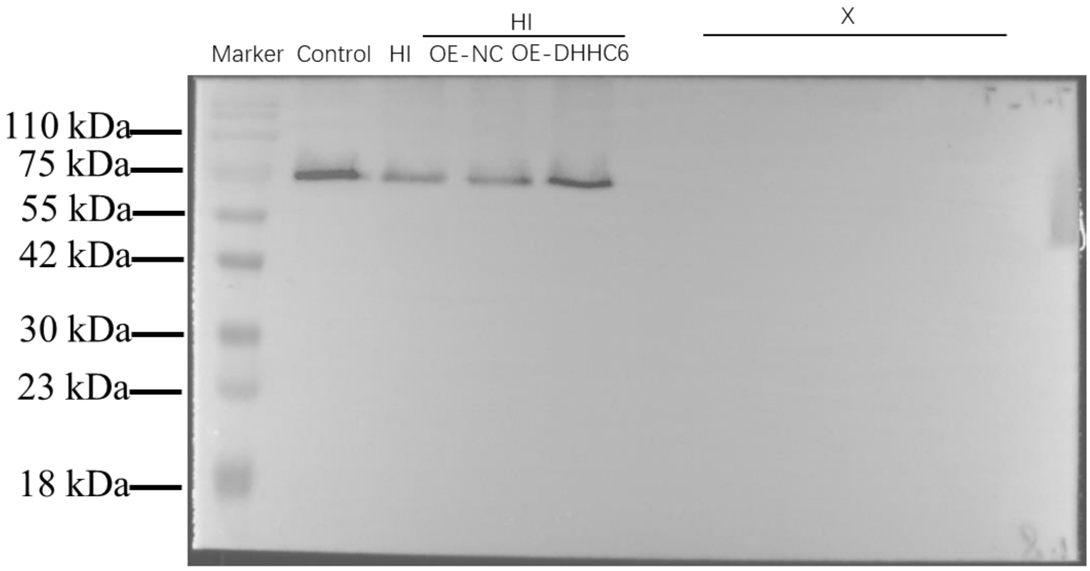

GRP75 -75 kDa

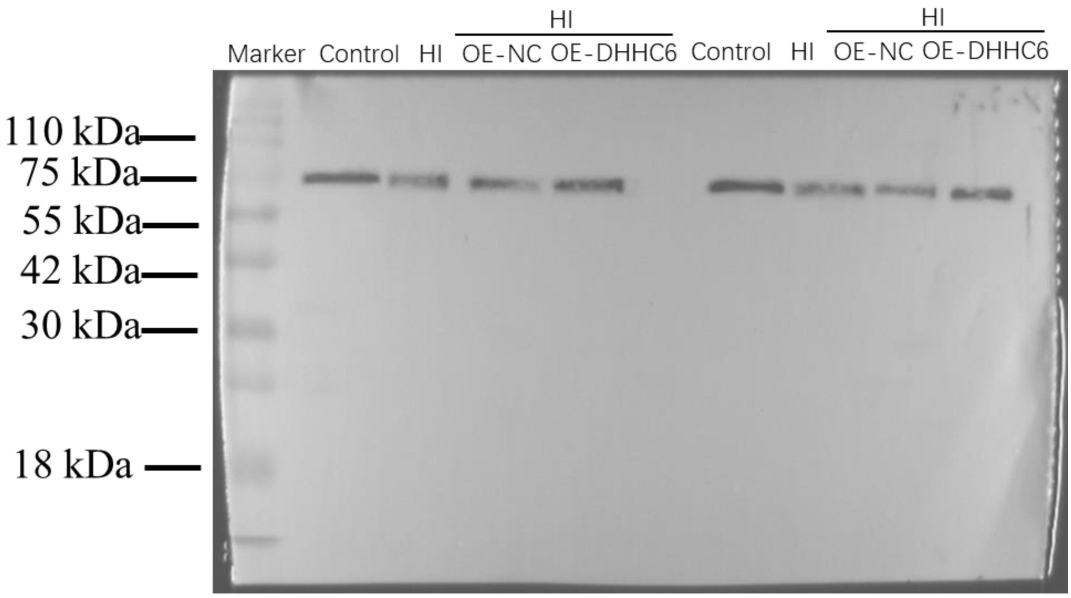

GRP75 -75 kDa

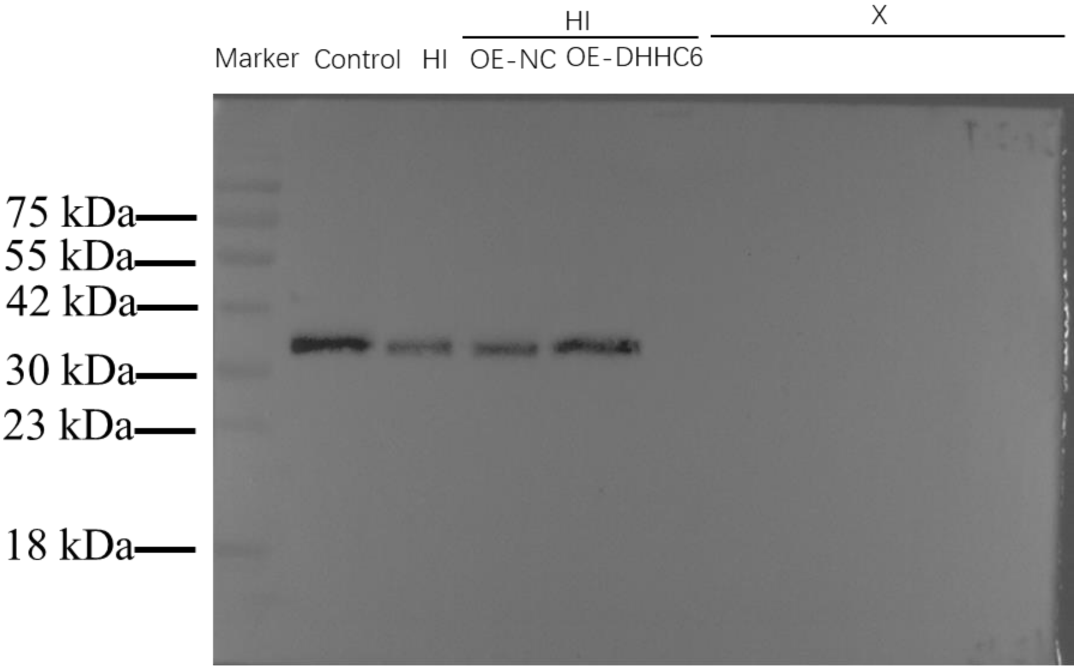

VDAC1 -33 kDa

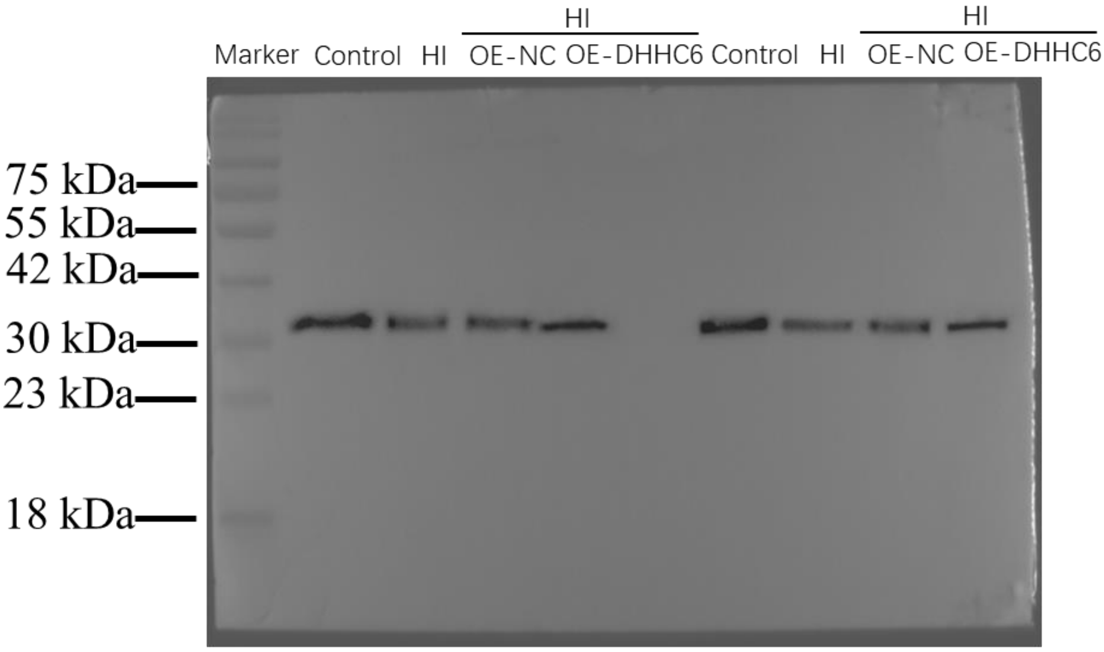

VDAC1 -33 kDa

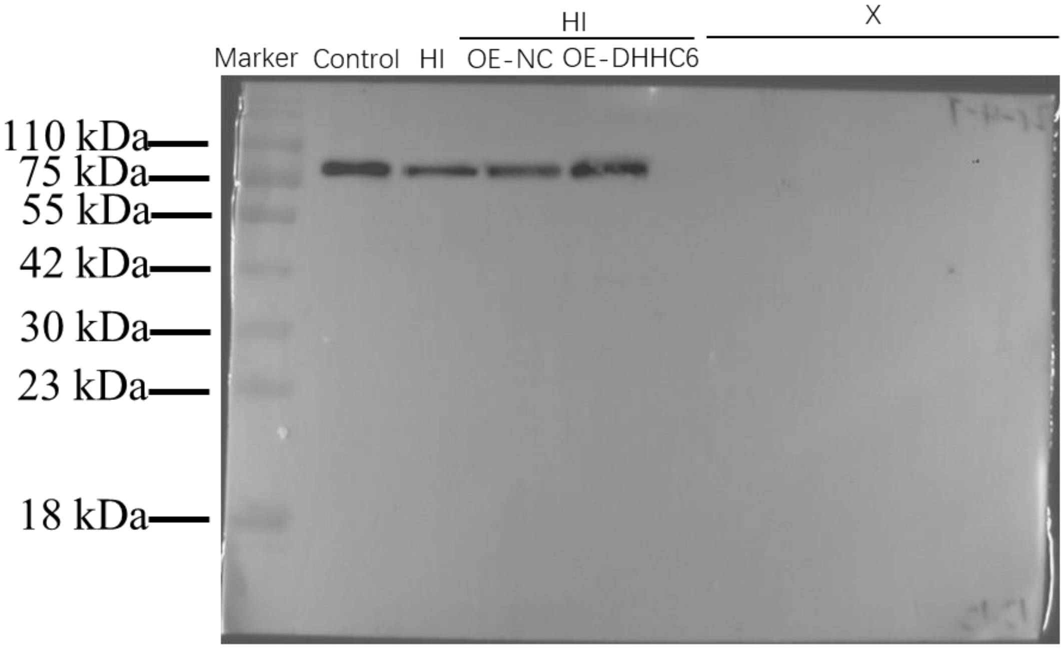

Drp1 -83 kDa

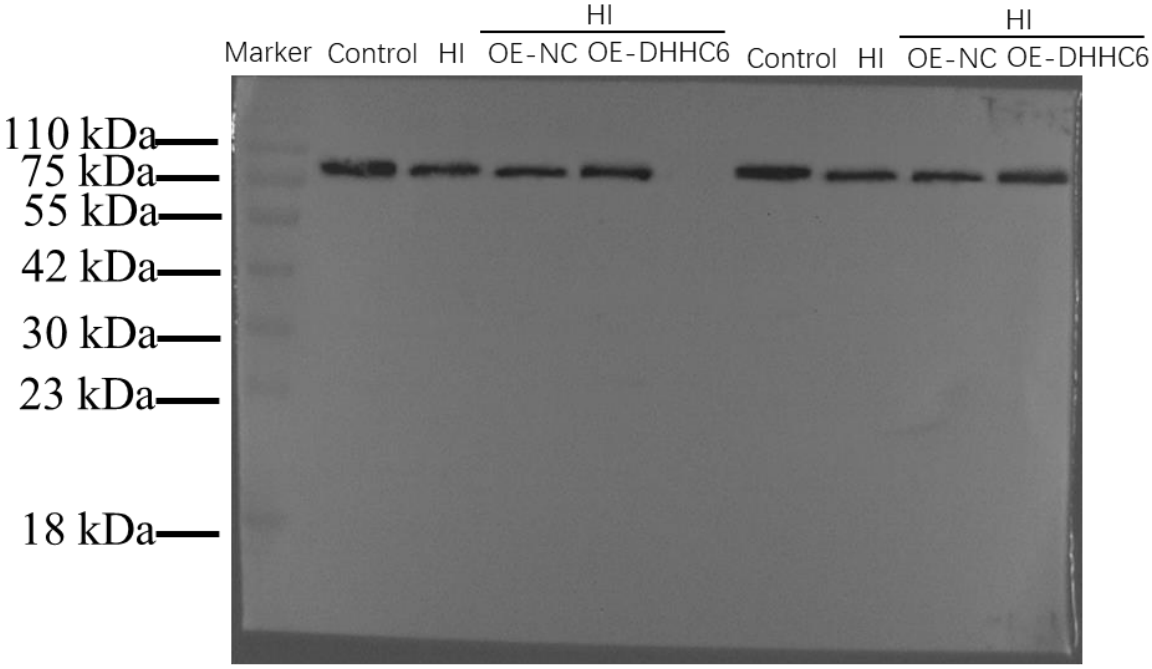

Drp1 -83 kDa

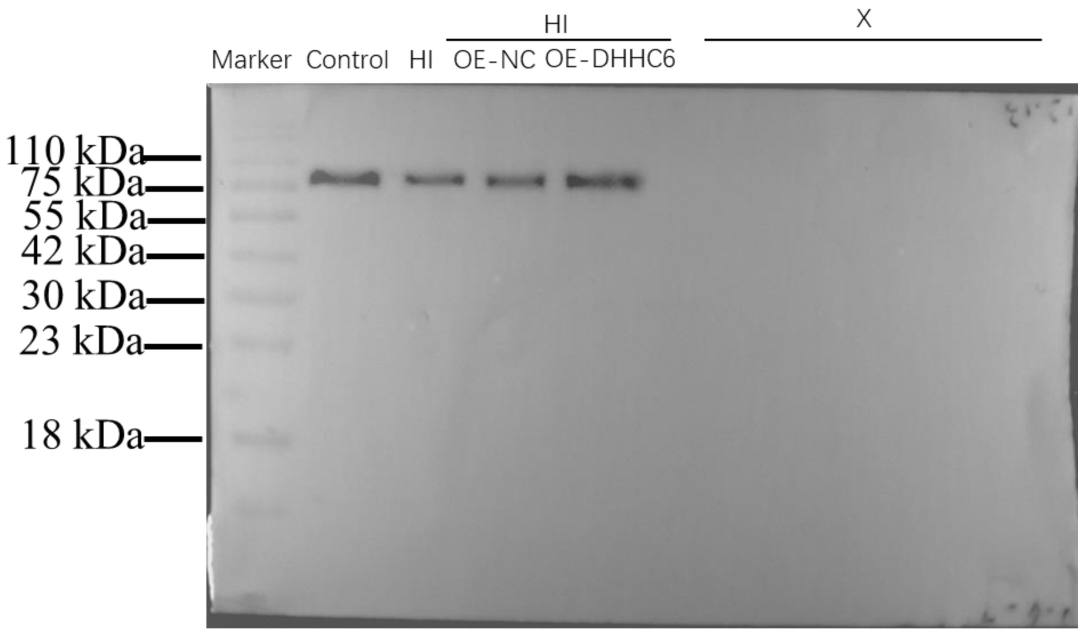

Mfn2 -86 kDa

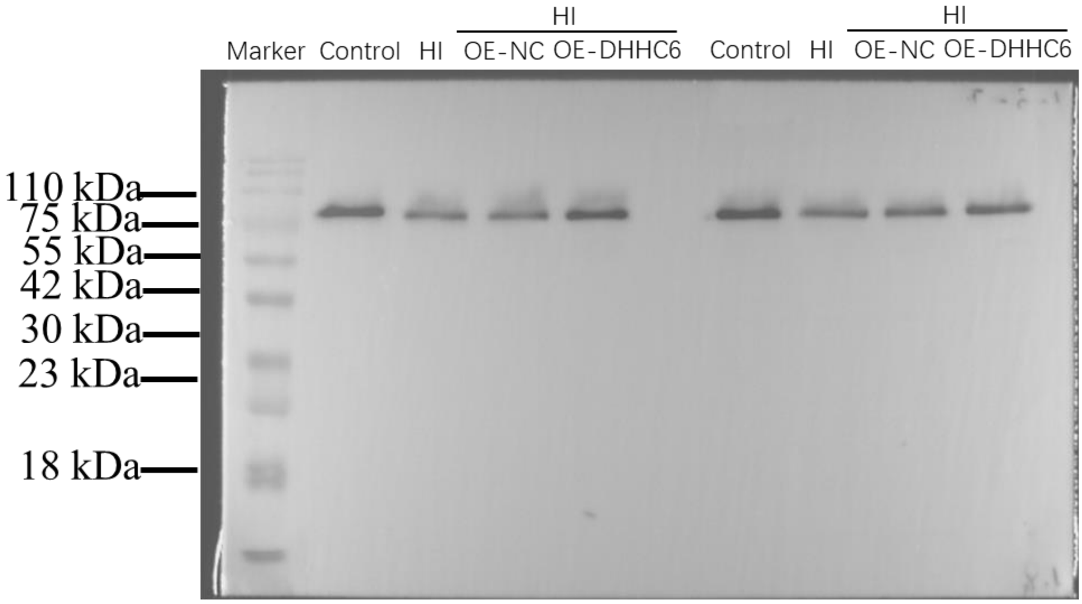

Mfn2 -86 kDa

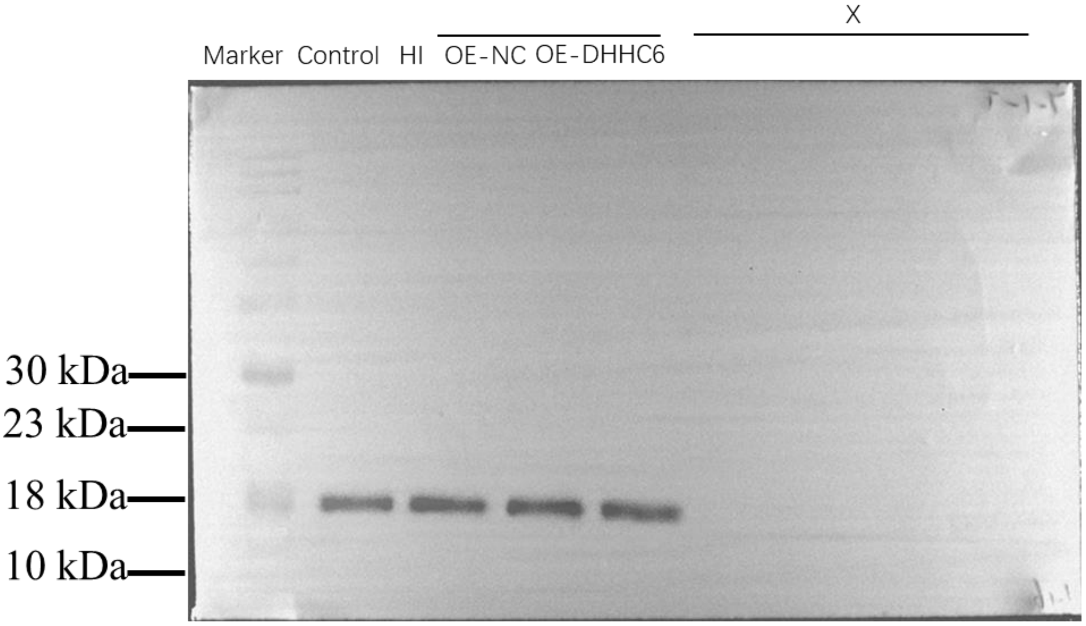

COX IV -17 kDa

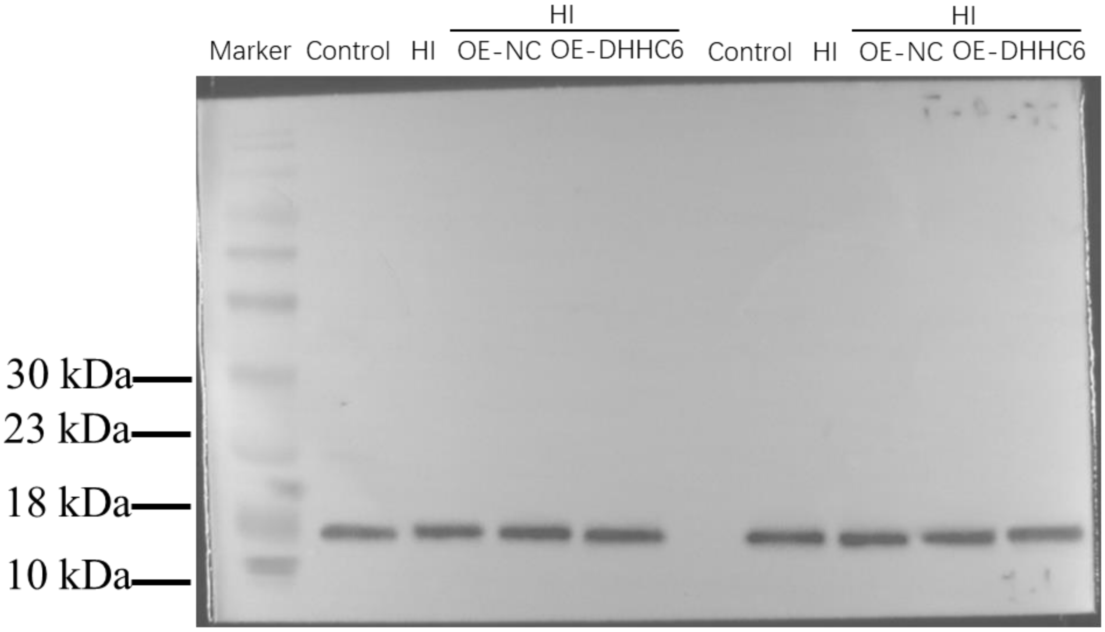

COX IV -17 kDa

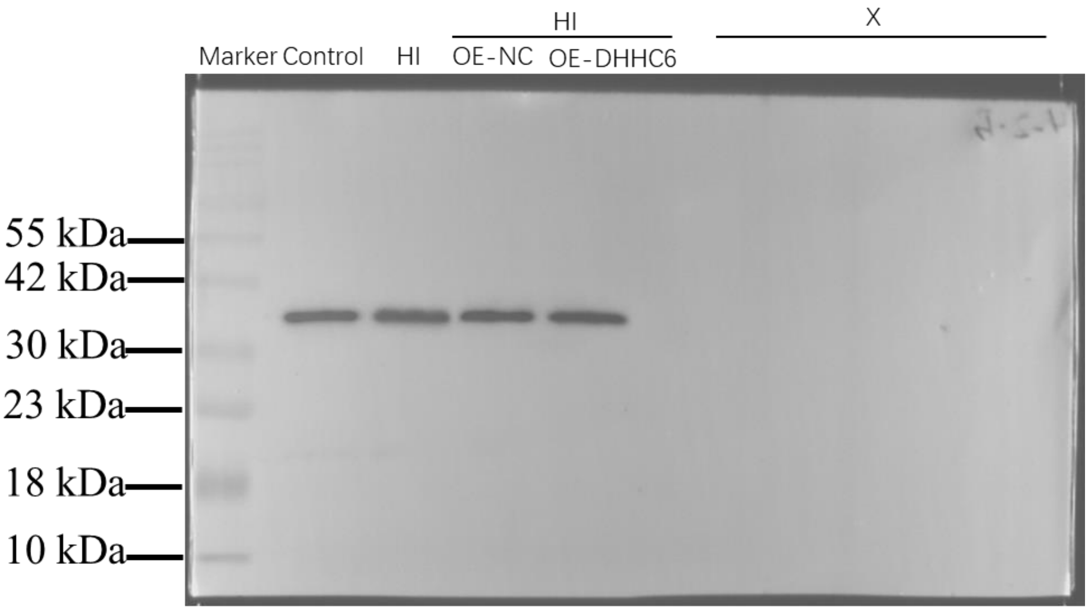

GAPDH-36 kDa

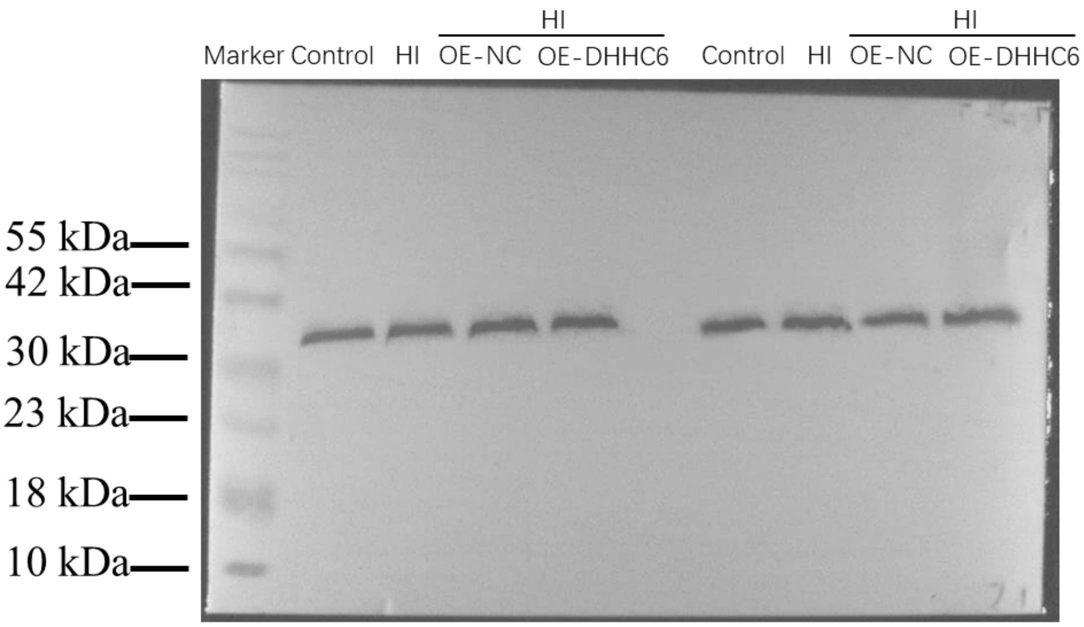

GAPDH-36 kDa

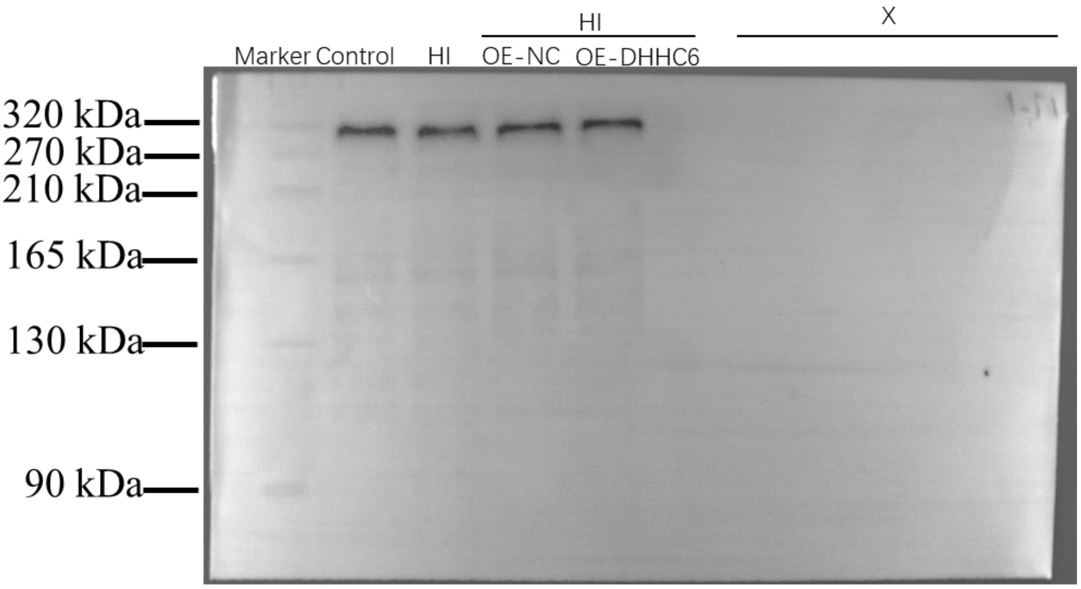

IP3R-314 kDa

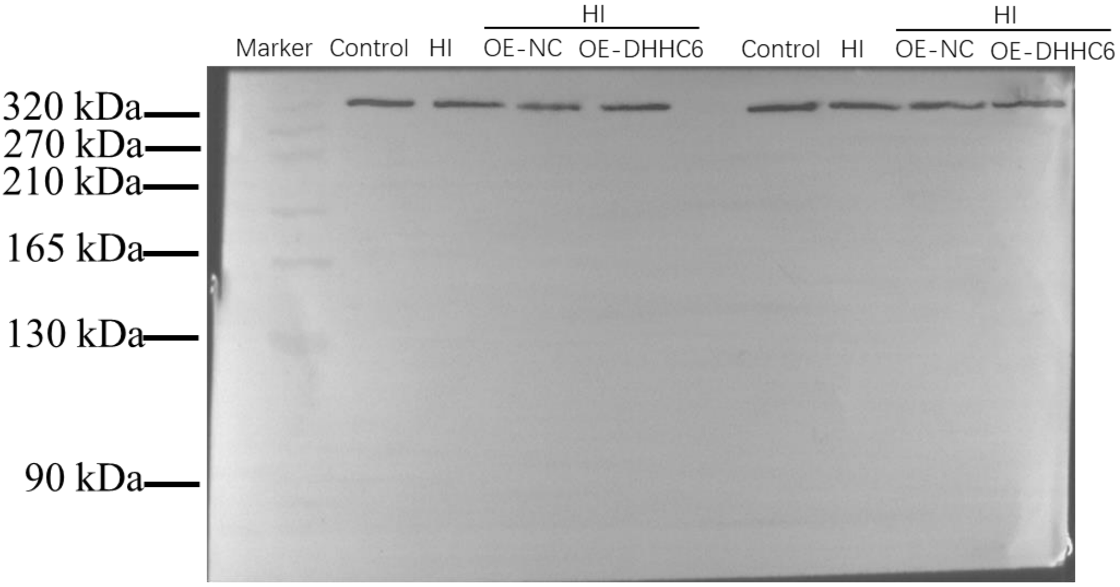

IP3R-314 kDa
